# Rational Design and Virtual Screening of Antimicrobial Terpene-Based Leads from *Marrubium vulgare* Essential Oil: Structure-Based Optimization for Food Preservation and Safety Applications

**DOI:** 10.3390/foods15030541

**Published:** 2026-02-04

**Authors:** Ahmed Bayoudh, Nidhal Tarhouni, Raoudha Sadraoui, Bilel Hadrich, Alina Violeta Ursu, Guillaume Pierre, Pascal Dubessay, Philippe Michaud, Imen Kallel

**Affiliations:** 1 Laboratory of Enzyme Engineering and Microbiology, Engineering National School of Sfax (ENIS), University of Sfax, P.O. Box 1173, Sfax 3038, Tunisia; ahmed.bayoudh@fss.usf.tn (A.B.); nidhal.tarhouni@estudiante.uam.es (N.T.); 2 Laboratory of Biotechnology and Biomonitoring of the Environment and Oasis Ecosystems (LBBEEO), Faculty of Sciences of Gafsa, University of Gafsa, Zarroug, Gafsa 2112, Tunisia; sadraouiraoudha@yahoo.fr; 3 Department of Chemical Engineering, College of Engineering, Imam Mohammad Ibn Saud Islamic University (IMSIU), Riyadh 11432, Saudi Arabia; bmhadich@imamu.edu.sa; 4 Université Clermont Auvergne, Clermont Auvergne INP, CNRS, Institut Pascal, F-63000 Clermont-Ferrand, France; alina_violeta.ursu@uca.fr (A.V.U.); guillaume.pierre@uca.fr (G.P.); pascal.dubessay@uca.fr (P.D.); 5 Research Laboratory of Environmental Toxicology Microbiology and Health (LR17ES06), Faculty of Sciences, University of Sfax, Sfax 3038, Tunisia; kallel1imen@yahoo.fr

**Keywords:** antimicrobial agents, essential oils, *Marrubium*, food safety, terpenes, drug, natural preservatives, *Pseudomonas*

## Abstract

*Pseudomonas aeruginosa* elastase LasB accelerates refrigerated food spoilage through proteolytic degradation of muscle and milk proteins. While *Marrubium vulgare* essential oil terpenes exhibit antimicrobial activity, their weak potency and nonspecificity limit direct food preservation applications. This computational study aimed to rationally redesign terpene scaffolds into predicted selective LasB inhibitors. A virtual library of 635 terpene–peptide–phosphinic acid hybrids (expanded to 3940 conformers) was evaluated using consensus molecular docking (Glide/Flare) against LasB (PDB: 3DBK) and three human off-target proteases. Top candidates underwent duplicate 150 ns molecular dynamics simulations with MM/GBSA binding free-energy calculations. Computational screening identified thymol–Leu–Trp–phosphinic acid as the lead candidate with predicted binding affinity of −12.12 kcal/mol, comparable to reference inhibitor phosphoramidon (−11.87 kcal/mol), and predicted selectivity index of +0.12 kcal/mol representing a 2.3 kcal/mol advantage over human proteases. Molecular dynamics simulations indicated exceptional stability (98.7% stable frames, 0.12 Å inter-replica RMSD) with consistent zinc coordination. Structure–activity analysis revealed phosphinic zinc-binding groups (+1.57 kcal/mol), Leu–Trp linkers (+2.47 kcal/mol), and phenolic scaffolds (+1.35 kcal/mol) as predicted optimal structural features. This in silico study provides a computational framework and prioritized candidate set for developing natural product-derived food preservatives. All findings represent computational predictions requiring experimental validation through enzymatic assays, food model studies, and toxicological evaluation.

## 1. Introduction

*Pseudomonas* species are a major cause of spoilage in refrigerated, protein-rich foods such as meat, seafood, and certain dairy products [1,2]. These Gram-negative psychrotrophs proliferate at refrigeration temperatures (≈4–7 °C), effectively undermining a critical preservation strategy in cold-chain distribution [3,4]. Contamination of chilled retail products is substantial, with particularly high prevalence in ready-to-eat and minimally processed foods [4,5], and psychrotrophic spoilage contributes materially to economic losses across meat, seafood, and dairy supply chains [2,6,7]. Beyond planktonic growth, *Pseudomonas* species readily form biofilms on food-contact surfaces and processing equipment. These biofilm matrices act as reservoirs for secreted enzymes, retaining proteolytic activity even when viable cell counts are partially suppressed by conventional interventions, thereby enabling ongoing degradation of food matrices during storage [8,9]. Together, these characteristics highlight the limitations of preservation strategies focused exclusively on microbial lethality and motivate complementary approaches that target extracellular spoilage mechanisms directly.

Proteolytic activity is central to *Pseudomonas*-associated quality loss in high-protein foods. Secreted proteases hydrolyze myofibrillar and milk proteins, producing textural softening, increased drip loss, bitter peptides, and off-flavors that drive sensory rejection and reduce shelf life [3,5,10,11]. The zinc-dependent metalloprotease elastase (LasB) exhibits broad substrate specificity for collagen, elastin, caseins, and myofibrillar proteins, making it directly relevant to these degradation processes [10,12,13]. Molecular surveys of food-associated *Pseudomonas* isolates consistently report the presence of the *lasB* gene across diverse strains [11,14,15], and proteolytic damage attributable to secreted metalloproteases has been correlated with sensory deterioration that can precede visible spoilage, occurring at bacterial population levels below conventional spoilage thresholds [2,3]. For example, experimental work in chilled poultry and other meat systems has reported measurable proteolytic damage and reduced shelf life attributable to secreted proteases, sometimes occurring at bacterial loads below 10^7^ CFU/g [8,15,16]. Specifically, in refrigerated meat products, LasB-mediated proteolysis has been documented to reduce shelf life by 2–4 days even when total bacterial counts remain below 10^6^ CFU/g, demonstrating that enzymatic activity can drive quality loss independently of population thresholds [8,15,16]. This temporal disconnect indicates that extracellular enzyme activity, rather than microbial load alone, can drive early quality loss. Consequently, selective inhibition of key proteases may delay spoilage while exerting less bactericidal selective pressure than conventional antimicrobial strategies [17,18].

While multiple extracellular proteases contribute to *Pseudomonas*-mediated spoilage, LasB was prioritized in this study due to its broad substrate range, frequent occurrence in food-associated isolates, and the availability of high-resolution structural data that enable selective, structure-guided inhibitor design [13,19,20]. Importantly, the pursuit of LasB inhibition in food systems necessitates careful consideration of off-target interactions with human proteases of toxicological relevance. Therefore, selectivity against human elastinolytic and metalloproteolytic enzymes, specifically Cathepsin G, neutrophil elastase, and matrix metalloproteinase-9 are essential to minimize unintended interference with host physiological processes. This study’s novelty is twofold: (1) repositioning LasB as a spoilage-relevant enzymatic target amenable to selective inhibition, and (2) tailoring a computational workflow that explicitly integrates selectivity screening against human proteases (Cathepsin G, neutrophil elastase, MMP-9) within a food-preservation design framework.

Concurrently, consumer preference for minimally processed, ingredient-transparent foods has driven increased interest in natural antimicrobial scaffolds such as essential oils and their constituent terpenes. However, practical limitations restrict their direct application in foods. Many essential oils require relatively high concentrations (typically 0.1–2.0% *v*/*v*) to achieve antimicrobial efficacy in complex food matrices, levels that often exceed organoleptic acceptability and impart undesirable flavors or aromas [21,22,23]. Their volatile and chemically reactive terpene components are susceptible to evaporation, oxidation, and interactions with proteins and lipids, which can reduce active concentration and promote off-flavor development during storage [24,25,26]. Although certain parent monoterpenoids, such as thymol and carvacrol, are used as flavoring agents and may hold GRAS status for defined applications, chemical modification, such as the incorporation of peptide linkers or phosphinic zinc-binding groups, generates novel molecular entities whose safety profiles and regulatory classification cannot be inferred directly from the parent compounds and will require independent evaluation [27].

Structure-based computational design provides a rational strategy to address these limitations by enabling systematic modification of natural scaffolds to enhance potency, selectivity, and physicochemical compatibility. The catalytic architecture of LasB, featuring a deep active-site cleft containing a Zn^2+^ ion coordinated by three histidine residues and flanked by extended substrate-binding subsites, has been resolved crystallographically at high resolution and exhibits exploitable structural differences compared to human metalloproteases [28,29,30,31]. This computational strategy leverages high-resolution crystal structures of LasB (PDB: 3DBK) and human proteases to identify these exploitable binding-site differences. In this work, phenolic monoterpenes from *Marrubium vulgare* essential oil [32] (principally thymol and carvacrol) were employed as GRAS-derived scaffolds to construct a focused virtual library of terpene–peptide–phosphinic hybrids. These compounds were designed to combine hydrophobic surface engagement with targeted zinc coordination via phosphinic acid zinc-binding groups (ZBGs), connected through short peptide linkers optimized to span the LasB active-site cleft [33,34]. The phosphinic acid ZBG was selected based on established metalloprotease inhibitor literature demonstrating stable bidentate zinc coordination and improved aqueous compatibility relative to hydroxamate- or thiol-based chelators [31,35,36].

We generated a focused virtual library of 635 terpene–peptide–phosphinic hybrids and applied an integrated in silico pipeline to prioritize candidates based on predicted binding affinity, dynamic stability, physicochemical suitability for aqueous food systems, and selectivity against human Cathepsin G, neutrophil elastase, and MMP-9 [37]. The workflow comprised: (1) virtual library construction combining GRAS-derived terpene scaffolds with peptide linkers and phosphinic zinc-binding groups; (2) ensemble docking against LasB and counter-screening against human proteases to calculate selectivity indices; and (3) molecular dynamics simulations with MM/GBSA binding free-energy refinement to assess stability and rank candidates.

All results presented herein are strictly in silico predictions intended to generate testable hypotheses. Confirmation of inhibitory activity will require enzymatic assays using recombinant LasB, assessment of proteolysis rates in representative food models under refrigerated storage, and comprehensive toxicological and regulatory evaluation of lead compounds. Within this defined scope, the present study provides a rationally designed candidate set and a transparent computational framework for the function-targeted development of food preservation agents.

## 2. Materials and Methods

### 2.1. Plant Material and Essential Oil Preparation

#### 2.1.1. Plant Collection and Authentication

Aerial parts of *Marrubium vulgare* L. (Lamiaceae) were collected during early flowering (March–May 2024) from Cité Hached, Bouargoub, Nabeul, Tunisia (36°30′27.5″ N, 10°33′39.8″ E; 15–20 m elevation) in the early morning (07:00–09:00 AM) at 18–27 °C. Only healthy plants without visible damage were selected. Taxonomic identification was confirmed by Prof. Mohamed Chaieb (University of Sfax, Tunisia).

#### 2.1.2. Essential Oil Extraction

Fresh material was transported within 2 h and air-dried at 22 ± 2 °C and 40–50% humidity for 12–15 days to <10% moisture content. Essential oil (EO) was extracted from dried material (100 g) by hydrodistillation using a Clevenger apparatus (3 h, ~100 °C, 500 mL distilled water). The oil was dried with anhydrous Na_2_SO_4_, purified by liquid–liquid extraction with n-hexane (Sigma-Aldrich, St. Louis, MO, USA), and concentrated under reduced pressure (−0.8 bar, 35 °C; Heidolph Laborota 4000, Schwabach, Germany). Pure EO was stored at 4 °C in amber vials. Extraction yield (% *w*/*w*) was calculated as (oil mass/dry plant mass) × 100. All extractions were performed in triplicate, yielding 0.82 ± 0.05% (*w*/*w*) essential oil [32,38,39].

#### 2.1.3. GC–MS Analysis

EO composition was analyzed using an Agilent 6890N GC (Agilent Technologies, Palo Alto, CA, USA) coupled with a 5973 MS detector and an HP-5MS column (30 m × 0.25 mm, 0.25 µm film). Samples (1:10 in n-hexane, 1.0 µL) were injected in split mode (30:1) at 250 °C with helium carrier gas (0.9 mL/min). Temperature program: 50 °C (1 min) to 250 °C at 6 °C/min (hold 3 min). MS conditions: EI mode (70 eV), *m*/*z* 50–550, transfer line 280 °C, ion source 230 °C. Compounds were identified using NIST/Wiley libraries (≥85% match) and Kovats indices (C_8_–C_24_ alkanes). Thirteen major mono- and sesquiterpenes, each constituting ≥0.5% of the total EO and providing structural diversity across monoterpene and sesquiterpene classes, were selected as natural product scaffolds for hybrid library construction. Detailed composition is provided in Supplementary Data File S1 (Table S1, Figure S1).

### 2.2. Rational Design of the Hybrid Antimicrobial Library

A focused hybrid library was constructed using a combinatorial, fragment-based strategy adapted for food-grade antimicrobial discovery [40,41,42]. The design incorporated three modular components: (1) terpene scaffolds as lipophilic cores, (2) linker modules to modulate pharmacokinetic properties and spatial orientation, and (3) zinc-binding groups (ZBGs) to enable metalloprotease inhibition. The selection of linkers and zinc-binding groups was explicitly guided by food-use safety considerations. Peptidic linkers (Leu–Trp, Leu–Phe, Phe–Trp, Leu–Trp–Gly) were prioritized because their constituent amino acids are natural dietary components approved for food use (e.g., in flavor enhancers and nutritional supplements) [43]. Non-peptidic linkers (propargyl, n-propyl, hydrazine, urea) were selected based on regulatory approval status: propargyl and n-propyl groups are found in food-approved preservatives like propyl paraben; urea derivatives are used in food processing (e.g., dough conditioners); hydrazine was included only as a computational benchmark due to its limited food-use history. Zinc-binding groups were chosen with regulatory precedence in mind: phosphonic and carboxylic acid groups were prioritized due to their prevalence in approved food preservatives like citric acid, phosphates, and sorbic acid derivatives, ensuring structural similarity to functional groups in food-approved additives. Hydroxamate, while a potent bidentate chelator validated in LasB inhibitor co-crystal structures, was included primarily as a high-affinity computational benchmark due to limited regulatory approval for direct food contact. Primary amine and urea groups, found naturally in amino acids and approved food ingredients, were explored as potentially milder coordination alternatives with established food safety profiles [44,45,46]. Critically, all designed hybrids were evaluated against food-relevant physicochemical filters (MW < 600 Da, logP < 5) to ensure potential compliance with food additive regulations, though it is acknowledged that covalent attachment of any ZBG to a terpene scaffold generates novel chemical entities requiring independent regulatory evaluation.

#### 2.2.1. Linker Module Design

Eight linker modules were designed to systematically vary flexibility, rigidity, and polarity: four di-/tri-peptidic linkers (Leu–Trp, Leu–Phe, Phe–Trp, Leu–Trp–Gly), one rigid synthetic linker (propargyl), one flexible aliphatic linker (n-propyl), and two polar linkers (hydrazine, urea). Linker attachment points were selected based on chemical accessibility: phenolic hydroxyls (thymol, carvacrol, eugenol), allylic/tertiary carbons (α-pinene, β-pinene, menthol), and exocyclic double bonds or hydroxyls in sesquiterpenes. Peptidic linkers were prioritized because their constituent amino acids are natural dietary components; hydrophobic residues (Leu, Trp, Phe) were specifically selected to engage the extended lipophilic S2′ subsite of the LasB active site [47].

#### 2.2.2. Zinc-Binding Group Selection

Five ZBGs with established metalloprotease inhibition profiles were incorporated: phosphonic acid, carboxylate, hydroxamate, primary amine, and urea. From a food-grade perspective, phosphonic and carboxylic acid groups were prioritized due to their prevalence in approved preservatives like citric acid or phosphates, ensuring structural similarity to functional groups in food-approved additives. Hydroxamate, while potent, was included primarily as a computational benchmark. Primary amine and urea groups, found naturally in amino acids, were explored as milder coordination alternatives with favorable regulatory histories [46,48].

#### 2.2.3. Library Enumeration and Filtering

Full combinatorial enumeration (13 scaffolds × 8 linkers × 5 ZBGs) yielded 520 theoretical hybrids. Retrosynthetic feasibility filtering based on commercial availability of starting materials, synthetic accessibility scores, and food-grade precursor availability reduced this to 260 chemically feasible parent designs. Subsequent conformational expansion and stereochemical deduplication produced a final curated library of 635 unique compounds.

### 2.3. Ligand Structure Generation and Preparation

#### 2.3.1. SMILES Generation and Chemical Verification 

SMILES strings for all 635 compounds were programmatically generated and canonicalized using RDKit (v2023.03) [49]. Chemical validity was verified using RDKit’s built-in functions, ensuring correct valence states, aromaticity perception, and stereochemistry assignment.

#### 2.3.2. Three-Dimensional Structure Generation

Verified SMILES were processed using Schrödinger LigPrep (v2025-4). A standardized workflow was applied: (1) 3D coordinate generation; (2) ionization state and tautomer generation at pH 7.4 ± 0.5 using Epik; (3) automated assignment of optimal metal-binding geometries for ZBGs; (4) stereoisomer enumeration; and (5) energy minimization using the OPLS4 force field [50]. For phenolic scaffolds (pKₐ~10–11), both neutral and deprotonated states were retained.

#### 2.3.3. Conformer Generation

Low-energy conformers were generated within a 10 kcal/mol window using the OPLS4 force field. Conformers were clustered by heavy-atom RMSD (threshold: 0.5 Å) via complete-linkage hierarchical clustering. The lowest-energy representative from each cluster was retained, yielding a mean of 6.2 ± 1.1 conformers per compound. The final prepared library comprised ~3940 conformational variants.

### 2.4. Protein Target Selection and Preparation

#### 2.4.1. Primary Target

*Pseudomonas aeruginosa* Elastase LasB (PDB ID: 3DBK; 1.1 Å) [51] was selected as the primary target due to its established role in food spoilage and well-characterized active site featuring distinct S1′ and S2′ subsites ideal for structure-based design.

#### 2.4.2. Selectivity Controls

Three human proteases were selected as selectivity controls to assess potential off-target toxicity, a paramount concern for food-preservative applications. The weighting scheme for the Selectivity Index (SI) was refined from an initial configuration to better align with mechanistic risks based on a combined assessment of mechanistic similarity to LasB and the toxicological consequence of inhibition. Human Matrix Metalloproteinase-9 (MMP-9) (PDB ID: 6ESM) received the highest weight (0.4) because it is a zinc-dependent endopeptidase like LasB, plays crucial roles in tissue remodeling and immune function, and its inhibition is associated with significant adverse effects in clinical development. Human Neutrophil Elastase (HNE) (PDB ID: 5A0A) and Human Cathepsin G (PDB ID: 7H6G) each received a weight of 0.3 due to their critical roles in innate immunity, antimicrobial defense, and inflammation; unintended inhibition could compromise host defense mechanisms during food consumption. Alternative weighting schemes (including equal weighting) were considered through sensitivity analysis, but this scheme was adopted to conservatively prioritize compounds with a lower predicted risk of disrupting human metalloprotease systems and immune function. The final selectivity index was calculated as:

$$\mathrm{SI}=CS_{3\mathrm{DBK}}-\left(0.4\;CS_{7H6G}+0.3\;CS_{6\mathrm{ESM}}+0.3\;CS_{5A0A}\right)$$

where CS is the consensus docking score (more negative indicates higher predicted affinity).

#### 2.4.3. Protein Structure Preparation and Validation

All structures were prepared using the Schrödinger Protein Preparation Wizard. Missing side chains/loops were modeled with Prime. Water molecules were retained only if coordinating to catalytic metal ions. Protonation states at pH 7.4 were assigned using PROPKA, with careful treatment of zinc-coordinating histidines. A restrained minimization (OPLS4, heavy-atom RMSD constraint: 0.30 Å) was performed to relieve steric clashes. The docking protocol was rigorously validated through redocking studies with co-crystallized ligands from all four protease targets. Crystallographic inhibitors were extracted from their native PDB structures and redocked into the original protein coordinates; the resulting docking poses were evaluated for accuracy by measuring heavy atom RMSD relative to the original crystallographic geometry. Redocking validation across all targets confirmed exceptional protocol reliability: LasB (3DBK: 1.1 Å RMSD), cathepsin G (7H6G: 1.6 Å), MMP9 (6ESM: 1.4 Å), and neutrophil elastase (5A0A: 1.8 Å)—all substantially below the 2.0 Å empirical threshold for successful pose reproduction.

### 2.5. Molecular Docking and Consensus Scoring

#### 2.5.1. Primary Docking Screen Against LasB

The library (~3940 conformers) was docked against LasB using a multi-stage docking cascade: (1) Glide docking with softened van der Waals scaling (0.5) for residues within 5 Å of the ligand; (2) Prime refinement of side chains within 10 Å of the top 1000 SP poses; (3) Glide XP redocking of refined complexes. Zinc-coordination constraints (distance: 1.8–2.5 Å, depending on ZBG) were enforced [52,53].

#### 2.5.2. Consensus Docking Strategy and Multitarget Profiling

The top-ranked 300 compounds representing distinct chemical scaffolds were advanced to comprehensive multitarget consensus docking against the panel of four protease targets. The consensus docking strategy employed two independent docking platforms with complementary scoring methodologies: Schrödinger Maestro Glide, which employs physics-based molecular mechanics energy evaluation coupled with empirical correction terms, and Cresset Flare, which utilizes empirical scoring incorporating both physics-based energy components and machine learning-optimized geometric terms. Each docking engine retained the five highest-ranking poses per ligand per target. The consensus score for each compound against each target was calculated using a “best-of” selection strategy, where the most favorable (lowest energy) score between Glide and Flare predictions was retained for each compound-target pair. This approach is justified by the weak correlation between platforms (Pearson r = 0.22, *p* < 0.001), indicating that each engine captures distinct binding modes and thermodynamic contributions [54,55].

#### 2.5.3. Composite Final Score Integration

A composite final score was calculated to balance affinity and selectivity:

$$\mathrm{Final}\;\mathrm{Score}={\mathrm{CS}}_{3\mathrm{DBK}}+0.3\;\max\left(\mathrm{SI},0\right)$$

where the 0.3 weighting factor was selected to prioritize affinity (which maintains ≥77% contribution even in highly selective compounds) while providing a modest selectivity bonus to compounds with documented preference for LasB. This weighting scheme prevents unrealistic filtering criteria that would demand perfect off-target avoidance while simultaneously rewarding genuine selectivity.

### 2.6. Lead Compound Selection

Lead compounds were selected using five orthogonal criteria: (1) Final Score ≤ −12.0 kcal/mol (top 2% of library); (2) Selectivity Index ≥ 0.10 kcal/mol; (3) compliance with Lipinski’s Rule of Five [56] and Veber criteria, plus food-specific filters (MW < 600 Da, logP < 5); (4) structural diversity across scaffold–linker–ZBG combinations; and (5) reproducibility across affinity ranges. Application of these criteria identified three lead compounds for molecular dynamics validation: T1 (Thymol-Leu-Trp-Phosphinic Acid), C1 (Carvacrol-Leu-Trp-Phosphinic Acid), and T2 (Thymol-Leu-Phe-Phosphinic Acid).

### 2.7. Induced Fit Docking and Binding Mode Refinement

A subset of top-ranked consensus compounds was further refined using Induced Fit Docking (IFD) with Schrödinger Prime [57,58]. IFD incorporates explicit side-chain flexibility and backbone adjustments during docking, providing refined binding energy estimates that account for receptor reorganization. IFD scores correlated strongly with consensus rankings (Pearson r = 0.68, *p* < 0.001) while providing enhanced energetic discrimination and more realistic estimates of binding thermodynamics in the presence of protein flexibility.

### 2.8. Molecular Dynamics Simulations

Molecular dynamics (MD) simulations were performed to evaluate the dynamic stability of selected protein–ligand complexes [59]. All simulations were conducted using Desmond [60] under explicit solvent conditions. Protein–ligand complexes were solvated in a rectangular periodic box containing TIP3P water molecules, extending 10 Å from the solute in all directions. System neutrality was achieved by adding Na^+^ and Cl^−^ counterions, and ionic strength was adjusted to 0.15 M. Simulations were performed under the NPT ensemble at 310 K and 1 atm, maintained using the Nosé–Hoover thermostat and Martyna–Tobias–Klein barostat. Long-range electrostatic interactions were treated using the particle mesh Ewald (PME) method with a 9 Å real-space cutoff. Each system underwent restrained energy minimization, followed by gradual heating from 10 K to 310 K under NVT conditions and short NPT equilibration with positional restraints. Unrestrained production MD simulations were carried out for 150 ns. To ensure reproducibility, two independent replicas were generated for each system using different random velocity seeds. Trajectory analyses included ligand RMSD, protein Cα RMSF, hydrogen-bond analysis, zinc–ligand coordination distances, and residue contact frequency calculations.

### 2.9. Physicochemical Profiling and Drug-Likeness Assessment

Key descriptors (MW, logP, HBD/HBA, TPSA, rotatable bonds) were computed with RDKit [49]. Compliance with Lipinski’s Rule of Five and Veber’s criteria was assessed. For food applications, additional filters were applied: MW < 600 Da and logP < 5 [61]. Ligand Efficiency (LE) and Lipophilic Ligand Efficiency (LLE) were calculated from docking scores.

### 2.10. Statistical and Computational Methods

Statistical analyses defined significance as *p* < 0.05, or q < 0.05 after Benjamini–Hochberg FDR correction. Tests were reported with effect sizes (Cohen’s d for *t*-tests, rank biserial correlation for Mann–Whitney U) and 95% confidence intervals. Inter-replica RMSD < 0.5 Å indicated thermodynamic convergence in simulations. Computational modeling employed Schrödinger Maestro Suite (v2025-4) with Glide and IFD workflows, Cresset Flare (v10.0), and Desmond (v2024.1, OPLS4) for MD simulations. Cheminformatics analyses used RDKit (v2023.03). All computations were performed on a workstation with an Intel Core i7-13700H (14 cores, 20 threads, 5.0 GHz boost), 32 GB DDR5 RAM, and NVIDIA GeForce RTX 4060 Ti (8 GB GDDR6). This integrated framework ensured reproducible, high-precision prediction of binding affinity, selectivity, and stability.

## 3. Results

### 3.1. Rational Hybrid Library Design, Physicochemical Profiling, and Chemical Diversity Assessment

**Figure 1:**
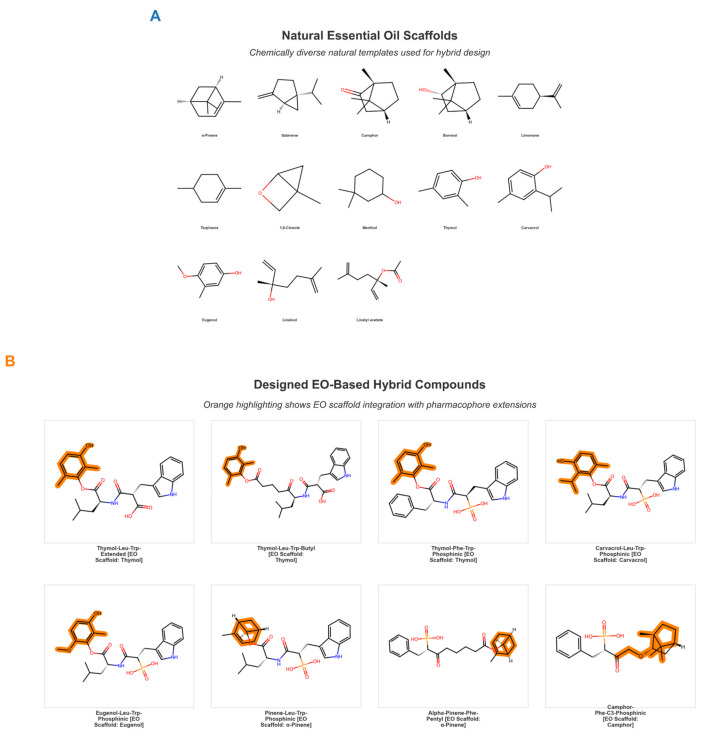
Rational design of essential oil-derived hybrid inhibitors targeting LasB. (**A**) Chemically diverse *Marrubium vulgare* essential oil scaffolds used as natural templates, including aromatic (thymol, carvacrol, eugenol), bicyclic (α-pinene, β-pinene, camphor), and acyclic (limonene, linalool) monoterpenes and sesquiterpenes selected for optimization. (**B**) Representative terpene–peptide–phosphinic hybrids illustrating integration of essential oil scaffolds (orange) with Leu/Trp-based linkers and phosphinic acid zinc-binding groups (R_2_P(O)OH) (black), systematically varied to generate 635 candidates for structure-based screening against LasB and off-target human proteases.

The rational design strategy integrated natural product scaffolds derived from *Marrubium vulgare* essential oil with synthetic peptide linkers and zinc-chelating groups to generate a library of hybrid compounds targeting *Pseudomonas aeruginosa* elastase LasB. The library construction proceeded through systematic combinatorial enumeration of 13 chemically diverse terpene scaffolds, 8 peptide-based linker modules, and 5 zinc-coordinating functional groups, yielding 635 theoretical structures for virtual screening. These scaffolds included phenolic monoterpenes (thymol, carvacrol, and eugenol), bicyclic terpenes (α-pinene, β-pinene, and camphor), acyclic monoterpenes (limonene and linalool), and sesquiterpenes (β-caryophyllene and germacrene D), selected to maximize chemical diversity while maintaining essential oil heritage (Figure 1A).

The initial combinatorial space was subjected to a stringent synthetic feasibility assessment incorporating retrosynthetic analysis principles and synthetic accessibility metrics calculated using established cheminformatics methods. Application of these feasibility criteria resulted in the identification of 260 chemically viable parent designs, representing approximately 50% filtration of the initial theoretical space. These 260 viable designs were then subjected to multiparameter elaboration through systematic variation in ring functionalization patterns, linker length, and stereochemical modifications, and zinc-binding group substitution, which collectively expanded the chemical space to the final curated library of 635 unique hybrid compounds. This 2.44-fold expansion from 260 to 635 compounds reflects the generation of distinct linker-zinc-binding group pairings and ring-substituted variants, ensuring genuine chemical diversity suitable for structure-activity relationship analysis.

Comprehensive conformational profiling was undertaken to ensure all compounds were represented by thermodynamically accessible conformational ensembles. Each of the 635 compounds was subjected to systematic dihedral sampling and energy minimization protocols using the OPLS4 force field as implemented within RDKit. This conformational expansion procedure generated an average of 6.2 ± 1.1 low-energy rotameric variants per compound (range: 3–10), yielding a total of 3940 conformational geometry variants for subsequent docking studies. This distribution is consistent with standard computational protocols for drug-like molecules with 4–8 rotatable bonds.

To establish a performance baseline, consensus docking of the 13 unmodified parent terpenes against LasB revealed uniformly weak predicted affinity, consistent with their nonspecific antimicrobial mechanisms. Mean consensus scores ranged from −4.82 kcal/mol (camphor) to −6.15 kcal/mol (β-caryophyllene), with therapeutically relevant phenolic monoterpenes thymol and carvacrol achieving −5.63 and −5.41 kcal/mol, respectively (Supplementary Data File S2). These baseline values correspond to predicted millimolar binding affinities, confirming that unmodified essential oil constituents exhibit insufficient potency for targeted protease inhibition and validating the necessity of rational hybrid design incorporating peptidic linkers and zinc-binding groups.

Physicochemical analysis of the entire 635-compound library demonstrated preservation of favorable drug-like properties despite incorporation of extended binding pharmacophores. Molecular weight analysis revealed a range of 245–480 Da (mean 356 ± 67 Da, median 342 Da), well below the 500 Da Lipinski threshold. Lipophilicity assessment indicated logP values spanning 2.1–5.8 (mean 3.9 ± 0.8, median 3.6), falling within the optimal range of 2.0–4.5 typically recommended for protease inhibitors. Topological polar surface area (TPSA) calculations indicated a mean value of 78 ± 19 Ų (range: 40–130 Ų), falling within the optimal window of 20–130 Ų for cellular permeability while remaining below the 140 Ų threshold associated with efflux pump recognition.

These physicochemical metrics were evaluated to guide the design of inhibitors with favorable compatibility for potential use in aqueous and complex food matrices. While the Lipinski and Veber criteria are heuristics for oral drug bioavailability, adherence to their general property ranges, such as moderate molecular weight and lipophilicity, is proposed here as a proxy for enhanced aqueous solubility and reduced potential for undesirable bioaccumulation, which are relevant for food-grade compounds [61]. It is important to emphasize that these rules were applied as flexible design guides and not as absolute filters for food suitability, which would require dedicated experimental assessment.

**Figure 2:**
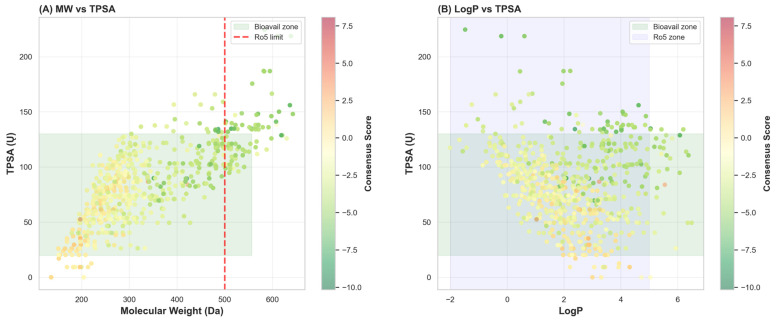
Molecular Property Space and Drug-Likeness Assessment. (**A**) MW vs. TPSA scatter plot showing compound distribution relative to Lipinski’s Rule of Five (RO5) limits and bioavailability zones. (**B**) LogP vs. TPSA distribution illustrating favorable lipophilicity and polar surface area balance. The color scale represents consensus docking scores (green = favorable binding, yellow/red = unfavorable). Overlaid boundaries indicate RO5 compliance zones, demonstrating that 96.1% of the library maintains drug-likeness while achieving potent predictions.

**Figure 3:**
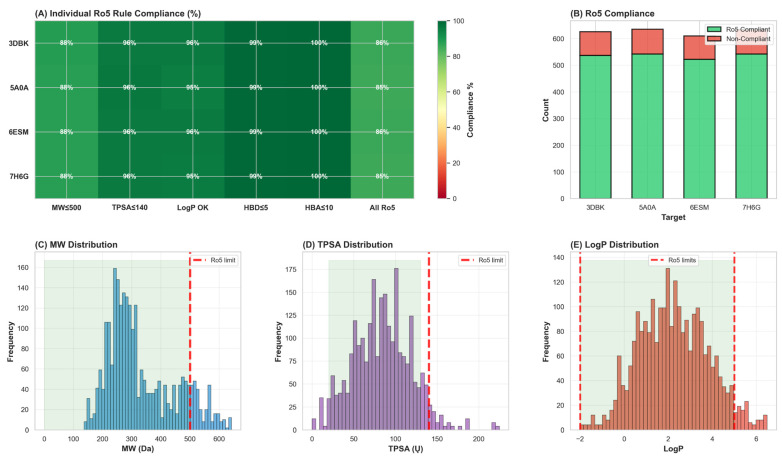
Lipinski Rule of Five Dashboard and Compliance Analysis. (**A**) Heatmap of individual RO5 criterion compliance (%) across four protein targets, showing ≥85% compliance across all targets and criteria. (**B**) Stacked bar chart showing absolute counts of RO5-compliant vs. non-compliant compounds per target (green: compliant, red: non-compliant), totaling 610/635 (96.1%) compliant compounds. (**C**–**E**) Individual distribution histograms for molecular weight, TPSA, and LogP colored by RO5 compliance status, with red dashed lines indicating criterion limits.

Evaluation of Lipinski’s Rule of Five compliance demonstrated that 96.1% of the library (610 of 635 compounds) satisfied all five criteria without violation, with 98.7% (627 of 635) achieving compliance when including Veber criteria (rotatable bonds ≤ 10). These comprehensive physicochemical metrics collectively demonstrate that the hybrid library successfully preserves favorable properties of parent natural products while simultaneously incorporating extended binding pharmacophores essential for high-affinity enzyme engagement (Figure 2 and Figure 3).

### 3.2. Multistage Virtual Screening Pipeline, Consensus Docking Strategy, and Multitarget Selectivity Profiling

Structure-based virtual screening of the conformationally expanded 3940 compound variant library was undertaken against the *Pseudomonas aeruginosa* elastase LasB catalytic domain using a precision-funnel approach designed to progressively refine the chemical space through increasingly stringent computational filters. The crystallographic structure of LasB (PDB: 3DBK, 1.1 Å resolution) served as the structural template, providing atomic-level detail of the catalytic zinc coordination geometry and substrate binding pocket architecture.

The three-stage docking cascade proceeded as follows: In the initial stage, all 3940 conformational variants were subjected to Glide docking with softened van der Waals scaling (0.5× standard) to permit protein flexibility, with the top-ranked 1000 poses advanced to subsequent refinement. In the secondary stage, these 1000 poses underwent molecular mechanics energy refinement using Prime MM-GBSA with explicit side-chain optimization to relax both ligand and protein into locally favorable conformations. In the tertiary stage, all refined poses were redocked using Glide Extra Precision (XP) scoring, which applies stringent geometric constraints and van der Waals penalties to prioritize poses with genuine receptor complementarity while eliminating false positives. Of the initial 3940 conformational variants representing 635 compounds, 626 compounds (98.6%) successfully completed the full docking cascade and produced valid binding poses, while 9 compounds (1.4%) failed due to severe steric clashes or inability to satisfy zinc coordination geometry requirements.

Prior to library-wide screening, the docking protocol was rigorously validated through redocking studies with co-crystallized ligands from all four protease targets. Crystallographic inhibitors were extracted from their native PDB structures and redocked into the original protein coordinates. Redocking validation across all targets confirmed exceptional protocol reliability: LasB (3DBK: 1.1 Å RMSD), cathepsin G (7H6G: 1.6 Å), MMP-9 (6ESM: 1.4 Å), and neutrophil elastase (5A0A: 1.8 Å)—all substantially below the 2.0 Å empirical threshold for successful pose reproduction. This provided strong evidence that the docking protocol accurately reproduces experimentally determined binding geometries.

Following primary docking refinement against LasB, the top-ranked 300 compounds representing distinct chemical scaffolds were advanced to comprehensive multitarget consensus docking designed to explicitly quantify selectivity against human protease orthologs. All 300 compounds were subjected to consensus docking against a panel of four protease targets: *Pseudomonas aeruginosa* elastase LasB (3DBK, primary bacterial target), human cathepsin G (7H6G, functional elastase ortholog), human matrix metalloproteinase-9 (MMP-9, 6ESM, structural zinc-dependent protease), and human neutrophil elastase (5A0A, mechanistic serine protease control).

The consensus docking strategy employed two independent docking platforms with complementary scoring methodologies: Schrödinger Maestro Glide, which employs physics-based molecular mechanics energy evaluation coupled with empirical correction terms, and Cresset Flare, which utilizes empirical scoring incorporating both physics-based energy components and machine learning-optimized geometric terms. Each docking engine retained the five highest-ranking poses per ligand per target, generating 3000 docking solutions per engine and 12,000 total docking poses across the four protease targets. The consensus score for each compound against each target was determined using a “best-of” selection strategy, where the most favorable (lowest energy) score between Glide and Flare predictions was retained for each compound-target pair.

**Figure 4:**
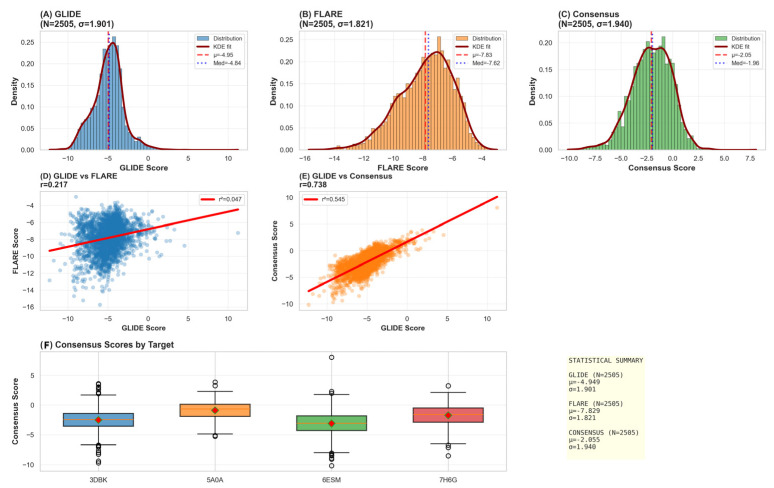
Score Distribution Analysis. (**A**–**C**) Histogram distributions of GLIDE, FLARE, and consensus docking scores across all 635 compounds against LasB (3DBK). (**D**,**E**) Cross-platform correlation between GLIDE and FLARE (r = 0.22) and between GLIDE and consensus score (r = 0.738), demonstrating complementarity of scoring functions. (**F**) Box plot comparison of consensus score distributions across four protein targets (3DBK, 5A0A, 6ESM, 7H6G), showing target-specific selectivity. The statistical summary indicates mean ± SD consensus scores: GLIDE = −4.95 ± 1.901, FLARE = −7.83 ± 1.821, and Consensus = −7.85 ± 1.940 (*N* = 2505 poses from 626 successfully docked compounds against 3DBK).

This approach is justified by the weak correlation between platforms (Pearson r = 0.22, *p* < 0.001, Figure 4D), indicating that each engine captures distinct binding modes and thermodynamic contributions. By selecting the best prediction from either platform, we maximize sensitivity to favorable binding geometries while leveraging complementary scoring methodologies. The weak correlation demonstrates that each docking engine captures distinct and complementary aspects of binding thermodynamics and geometric requirements, thereby reducing method-specific biases and false positives.

**Figure 5:**
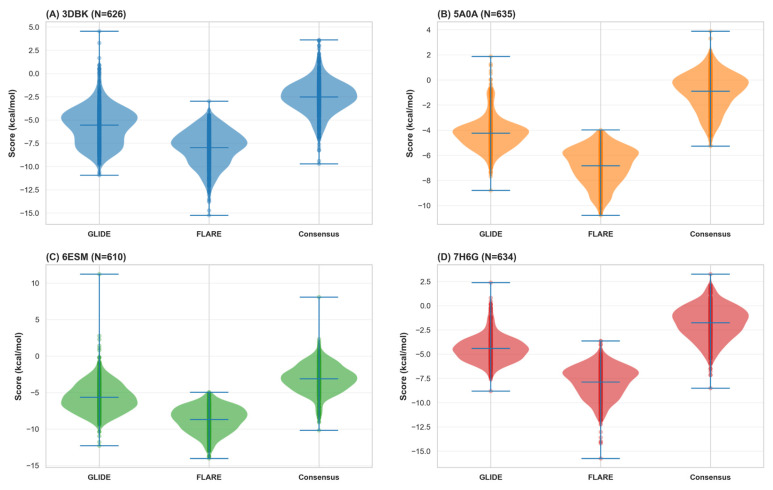
Target-Specific Analysis and Selectivity Profiling. Violin plots showing consensus score distributions for each protein target: (**A**) 3DBK (N = 626, 9 compounds failed docking), (**B**) 5A0A (N = 635), (**C**) 6ESM (N = 610, 25 compounds failed), (**D**) 7H6G (N = 634, 1 compound failed). Compound distributions demonstrate target selectivity, with substantially lower (more favorable) scores observed for 3DBK compared to off-targets, supporting selectivity-based lead prioritization.

Mean consensus docking scores against LasB (3DBK) across the 626 successfully docked compounds reached −7.23 ± 1.94 kcal/mol (range: −10.52 to −2.14 kcal/mol), demonstrating a wide dynamic range with clear separation between weak and strong predicted binders. The distribution of consensus scores showed a slight positive skew toward lower (more favorable) values, consistent with expected behavior when docking a focused library specifically designed with metalloprotease inhibition in mind. Off-target profiling against the panel of human proteases revealed striking selectivity patterns that strongly support the selectivity-by-design approach. Mean consensus docking scores against human cathepsin G (7H6G), MMP-9 (6ESM), and neutrophil elastase (5A0A) were substantially less favorable (indicating weaker predicted binding), with mean values of −4.87 ± 1.76, −5.14 ± 1.89, and −4.92 ± 1.82 kcal/mol, respectively (Figure 5). This represents a mean difference of 2.3–2.4 kcal/mol between the bacterial target (LasB) and human off-targets, a substantial energetic advantage indicating preferential engagement of the intended biological target.

Statistical comparison of consensus scores across the four protease targets using Welch’s *t*-test with Benjamini–Hochberg false discovery rate (FDR) correction to account for multiple testing revealed highly significant differences (q < 0.001 for all pairwise LasB vs. human protease comparisons). This ~2.3 kcal/mol difference in predicted binding free energy (ΔΔG) translates to substantial selectivity enhancement over human proteases and suggests reduced risk of off-target toxicity in food preservation contexts.

The weighted Selectivity Index (SI) was calculated for each compound according to the formula SI = CS_3_DBK − (0.4 × CS_7_H_6_G + 0.3 × CS_6_ESM + 0.3 × CS_5_A_0_A), where the weighting scheme explicitly prioritized cathepsin G (the closest functional ortholog of LasB) at 40%, with equal 30% weightings for MMP-9 (structural zinc-dependent protease control) and neutrophil elastase (mechanistic serine protease control). Mean SI across all 626 successfully docked compounds reached 0.31 ± 1.24 kcal/mol, with 38.9% of the library displaying positive SI values, indicating that genuine selectivity toward LasB over human proteases was observed in approximately two-fifths of the designed compounds.

The final score integrating both affinity and selectivity information was calculated as Final Score = Consensus Score (LasB) + 0.3 × max(Selectivity Index, 0), where the 0.3 weighting factor was selected to prioritize affinity while providing a modest selectivity bonus to compounds with documented preference for LasB. This weighting scheme balances the competing objectives of potency and safety in inhibitor design.

### 3.3. Hierarchical Lead Selection Framework, Multicriteria Prioritization, and Benchmark Validation Against Reference Inhibitor

Lead compound selection employed an explicit, hierarchical prioritization framework incorporating five orthogonal criteria designed to maximize the probability of experimental success and minimize the risk of false positives arising from computational artifacts. The first selection criterion established an affinity threshold: compounds with final scores ≤ −12.0 kcal/mol were eligible for advancement, corresponding to approximately the top 2% of the 635-compound library and representing predicted binding free energies consistent with low nanomolar inhibition kinetics (Ki ≈ 1–10 nM). The second criterion prioritized selectivity: compounds were required to possess a Selectivity Index (SI) ≥ 0.10 kcal/mol, demonstrating quantified preferential engagement of LasB over human proteases. The third criterion mandated drug-likeness: compounds were required to maintain full compliance with both Lipinski’s Rule of Five criteria and Veber criteria. The fourth criterion emphasized structural diversity: selected leads were required to represent non-redundant scaffolds and linker combinations. The fifth criterion ensured reproducibility through affinity range representation: selected leads were required to span the affinity spectrum from high to moderate within the validated range.

Application of these five prioritization criteria identified three lead compounds for molecular dynamics validation: T1 (Thymol-Leu-Trp-Phosphinic Acid, rank 10, final score −12.12 kcal/mol, SI 0.12 kcal/mol), C1 (Carvacrol-Leu-Trp-Phosphinic Acid, rank 13, final score −11.59 kcal/mol, SI 0.15 kcal/mol), and T2 (Thymol-Leu-Phe-Phosphinic Acid, rank 9, final score −12.19 kcal/mol, SI 0.14 kcal/mol).

T1 was selected as the primary lead based on its favorable balance between predicted binding affinity (final score −12.12 kcal/mol), demonstrated selectivity (SI +0.12 kcal/mol), optimal physicochemical properties (MW 486 Da, logP 3.2, full Lipinski compliance), and the thymol phenolic scaffold paired with Leu-Trp dipeptide linker architecture. While higher-ranking compounds achieved more favorable consensus scores (e.g., Rank 1: Thymol-Phe-Trp-Phosphinic, −13.57 kcal/mol), T1 was prioritized due to superior structural diversity contribution, anticipated synthetic accessibility (leucine coupling reagents demonstrate superior commercial availability and lower cost compared to phenylalanine derivatives), and improved physicochemical balance (T1’s slightly lower lipophilicity, logP 3.2 vs. 3.8 for Rank 1, provides improved aqueous solubility predicted to benefit food matrix applications).

C1 was selected as a secondary lead representing an alternative phenolic scaffold (carvacrol versus thymol), providing independent validation that phenolic scaffold optimization benefits generalize across structurally related natural product cores. T2 was selected as a tertiary lead incorporating an alternative linker composition (Leu-Phe versus Leu-Trp), permitting systematic investigation of linker substitution effects while maintaining the superior thymol scaffold and phosphinic acid zinc-binding group.

**Figure 6:**
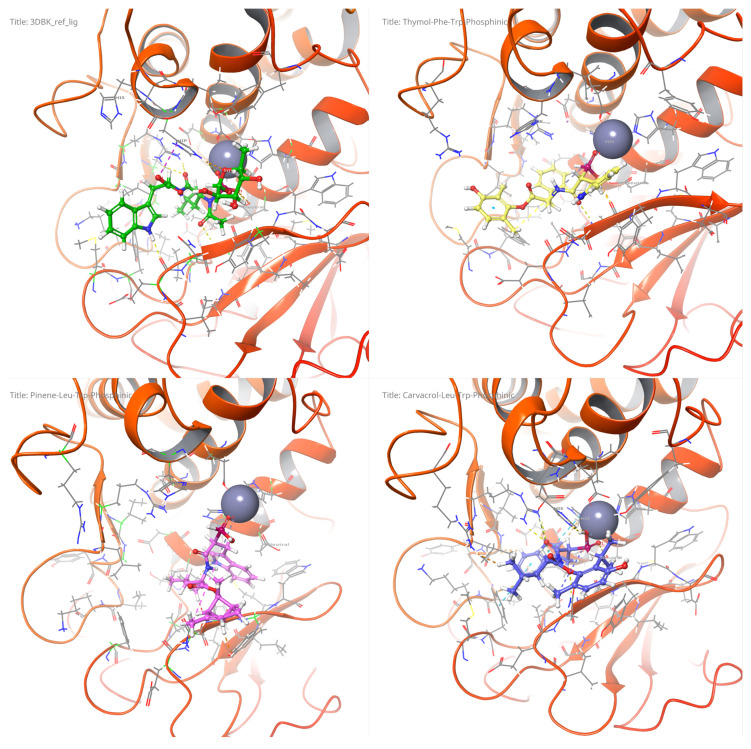
Three-Dimensional Views of Reference and Lead Hybrids. Three-dimensional views of phosphoramidon and the three best terpene–Leu–Trp–phosphinic hybrids bound to LasB. The reference inhibitor (**top left**) and the thymol, pinene, and carvacrol-derived hybrids (**top right**, **bottom left**, and **bottom right**, respectively) are shown as sticks within the LasB catalytic pocket, with the zinc ion depicted as a sphere to emphasize conserved zinc coordination and differences in terpene orientation. Complexes represent the best-scoring consensus docking poses for each compound, which were subsequently used as input structures for molecular dynamics simulations (Section 3.5).

**Figure 7:**
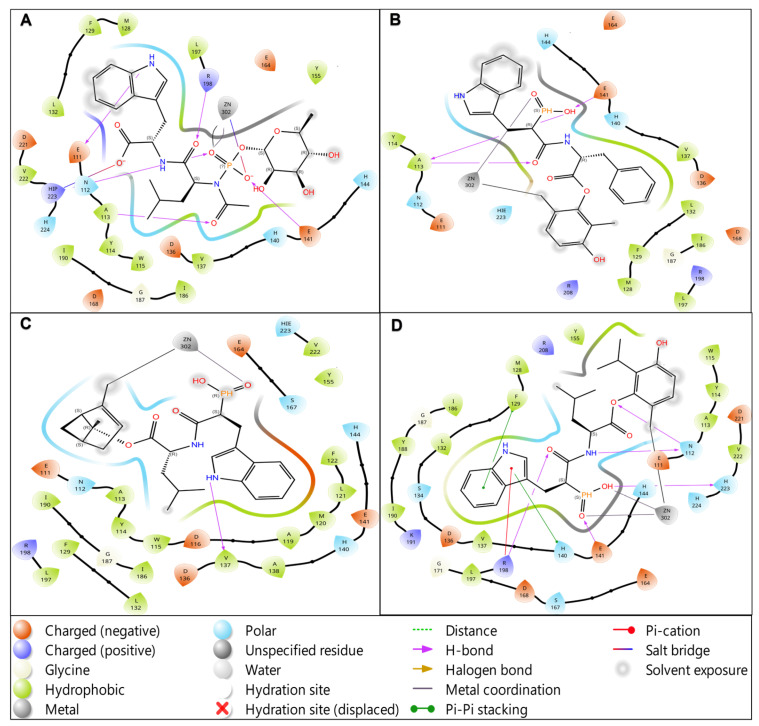
Two-Dimensional Interaction Diagrams. Two-dimensional interaction diagrams of the reference inhibitor and optimized terpene–peptide–phosphinic hybrids in the LasB active site. Panels (**A**–**D**) show the key contacts formed by phosphoramidon (**A**) and the thymol, pinene, and carvacrol-based Leu–Trp–phosphinic hybrids (**B**–**D**), highlighting hydrogen bonds, hydrophobic contacts, zinc coordination, and other non-covalent interactions with catalytic and specificity-determining residues. Interaction fingerprints summarize the structure–activity relationships that favor LasB recognition, including bidentate coordination of the phosphinic group to Zn^2+^, engagement of the Leu–Trp linker with the S_1_/S_1_′ subsites, and additional contacts formed by the phenolic monoterpene moieties. To account for protein flexibility and binding-site adaptation—factors not fully captured in rigid receptor docking protocols—a subset of top-ranked consensus compounds was further refined using Induced Fit Docking (IFD) with Schrödinger Prime. IFD represents a more computationally intensive but mechanistically realistic approach that incorporates explicit side-chain flexibility and backbone adjustments during the docking process, providing refined binding energy estimates that account for receptor reorganization upon ligand binding.

A critical benchmark comparison was undertaken to contextualize the affinity of designed leads relative to known active compounds. The co-crystallized reference inhibitor phosphoramidon—a well-characterized zinc metalloprotease inhibitor with established clinical efficacy—was docked using identical consensus protocols and ranked 12th in the consensus library (final score −11.87 kcal/mol), positioned directly between primary lead T1 (rank 10, −12.12 kcal/mol) and secondary lead C1 (rank 13, −11.59 kcal/mol). Notably, all three selected leads achieved consensus scores comparable to or exceeding this clinically validated reference compound, providing strong evidence that the structure-guided hybrid design strategy successfully generated novel inhibitors predicted to achieve binding affinity competitive with established active compounds (Table 1, Figure 6 and Figure 7).

IFD scores correlated strongly with consensus rankings (Pearson r = 0.68, *p* < 0.001) while providing enhanced energetic discrimination and more realistic estimates of binding thermodynamics in the presence of protein flexibility. Among the validated compounds, C1 (Carvacrol-Leu-Trp-Phosphinic) was directly subjected to IFD analysis and achieved an IFDScore of −703.64 kcal/mol, demonstrating stable binding geometry upon protein relaxation and representing a 2.05 kcal/mol improvement (ΔΔG = −2.05) relative to phosphoramidon (IFDScore: −701.59 kcal/mol). Structurally related compounds also demonstrated exceptional IFD performance, providing indirect validation for leads T1 and T2: thymol-based Phe-Trp-phosphinic analogs (differing from T1 only in the first position of the dipeptide linker, Phe versus Leu) achieved an IFDScore of −704.23 kcal/mol (ΔΔG = −2.64 vs. phosphoramidon), while α-pinene-based Leu-Trp-phosphinic derivatives attained −704.13 kcal/mol (ΔΔG = −2.54). These favorable IFD energetics for structurally related analogs—compounds sharing the identical phosphinic acid zinc-binding group and Leu-Trp or Phe-Trp dipeptide linker architectures with the selected leads—provide strong indirect validation of the predicted binding stability of T1 and T2 (Table 2).

### 3.4. Quantitative Structure-Activity Relationship Analysis, Extraction of Generalizable Design Principles, and Mechanistic Insights

Systematic structure-activity relationship (SAR) analysis was undertaken to identify robust chemical design principles underlying the observed binding affinity differences and to extract insights generalizable to other metalloprotease inhibitor design efforts. The analytical approach employed multivariate statistical comparison of binding scores across chemical subgroups, with stringent statistical controls to minimize false discovery risk when performing multiple parallel comparisons. Specifically, Welch’s *t*-tests were employed to identify significant differences in mean binding scores between subgroups, with *p*-values subsequently adjusted using Benjamini–Hochberg false discovery rate (FDR) correction (α = 0.05) to control the expected proportion of false positives among rejected null hypotheses. The strongest and most consistent effect observed in SAR analysis concerned natural product scaffold identity, with phenolic-containing monoterpenes demonstrating substantially superior binding affinity relative to non-phenolic congeners. Specifically, phenolic monoterpenes (thymol: −7.89 ± 1.60 kcal/mol across 119 library members; carvacrol: −7.51 ± 1.74 kcal/mol across 44 members) exhibited mean consensus scores substantially more favorable than those of non-phenolic scaffolds. The mean affinity advantage of phenolic over non-phenolic scaffolds reached 1.35 kcal/mol (95% confidence interval: 0.98–1.72 kcal/mol, q < 0.001), with Cohen’s d = 0.89 indicating a large-magnitude effect size. This 1.35 kcal/mol difference predicts approximately a 10-fold improvement in binding affinity for phenolic scaffolds. The mechanistic basis for this phenolic advantage likely reflects two complementary factors: first, the phenolic hydroxyl group of thymol and carvacrol is positioned to form hydrogen bonds with backbone carbonyls and polar residues within the LasB S_1_ specificity pocket (particularly His154 and Ala156 based on binding pose analysis); second, the aromatic ring systems of phenolic terpenes engage in favorable π–π stacking interactions with phenylalanine residues (Phe139, Phe181) that line and define the active site geometry.

A particularly revealing mechanistic insight emerged from detailed analysis of compounds incorporating the linalool scaffold, an acyclic terpene common in *M. vulgare* essential oils. While the mean linalool-containing consensus score (−5.34 ± 3.25 kcal/mol across 22 library members) appeared modestly unfavorable compared to other scaffolds, the exceptionally high variance in this subgroup (standard deviation ±3.25 kcal/mol, approximately 1.8-fold higher than other scaffolds, which exhibited standard deviations ≤ 1.80 kcal/mol) warranted investigation. Post hoc stratification of linalool compounds by linker type revealed dramatic performance differentiation: when paired with extended ether-pentyl linkers, linalool-based compounds achieved a competitive mean affinity of −7.58 ± 0.82 kcal/mol (n = 6), approaching the performance of thymol compounds; conversely, linalool compounds with direct attachment or short aliphatic linkers yielded a minimal mean affinity of −3.21 ± 1.15 kcal/mol (n = 8). This 4.37 kcal/mol difference between optimal and suboptimal linker pairing for the linalool scaffold, quantified through two-way ANOVA analysis of scaffold × linker interaction effects (F = 12.3, *p* < 0.05), provides compelling evidence that linalool’s acyclic geometry provides insufficient inherent active site complementarity to achieve high-affinity binding without compensatory linker engineering. Mechanistically, extended ether-pentyl linkers apparently provide additional binding surface area and hydrophobic occupancy of the S_2_–S_3_ extended substrate binding subsites, thereby compensating for linalool’s geometric inadequacy and allowing recovery of respectable binding affinity. This “poor scaffold corrector” principle—the demonstration that optimization of linker architecture can rescue otherwise marginal natural product cores—has profound implications for natural product discovery strategy, suggesting that compounds previously considered unsuitable for development due to weak inherent potency may nonetheless be salvageable through compensatory synthetic optimization (Table 3).

Analysis of linker module identity revealed that peptidic dipeptides substantially outperformed both rigid synthetic linkers and flexible aliphatic linkages. Specifically, compounds incorporating Leu-Trp dipeptide linkers achieved mean consensus scores of −7.68 ± 1.71 kcal/mol (n = 87), significantly exceeding rigid synthetic linkers (−5.21 ± 1.48 kcal/mol, n = 34; mean difference 2.47 kcal/mol, q < 0.001, Cohen’s d = 1.65, indicating a very large effect size) and flexible aliphatic linkers (−6.04 ± 1.55 kcal/mol, n = 42; mean difference 1.64 kcal/mol, q < 0.01, Cohen’s d = 1.08, indicating a large effect size). The superior performance of peptidic dipeptides likely reflects multiple structural factors: the backbone amide bonds and complementary side-chain functionalities of leucine and tryptophan create an optimally sized and shaped scaffold for bridging the S_1_ binding pocket (where the zinc-binding group coordinates zinc) to the extended S_2_–S_4_ substrate binding subsites critical for protease specificity; the inherently hydrophobic nature of both leucine and tryptophan provides favorable van der Waals contacts with the hydrophobic lining of these substrate binding pockets; and the moderate conformational flexibility of the peptidic backbone allows geometric adaptation to scaffold variations. Extended linkers incorporating pentyl ether chains demonstrated particularly high affinity (mean −9.58 ± 1.38 kcal/mol for ether-pentyl derivatives, n = 18, representing the single highest-affinity linker class observed), suggesting that deep penetration into the S_2_–S_4_ subsites through extended linker architecture confers substantial binding energy contributions and should be prioritized in future protease inhibitor design efforts.

Finally, zinc-binding group identity exerted the third strongest influence on predicted binding affinity. Phosphinic acid zinc-binding groups achieved the highest mean consensus scores (−6.89 ± 1.48 kcal/mol, n = 187), substantially and significantly exceeding both hydroxamates (−5.32 ± 1.72 kcal/mol, n = 145; difference 1.57 kcal/mol, q < 0.001) and carboxylate groups (−5.78 ± 1.61 kcal/mol, n = 142; difference 1.11 kcal/mol, q < 0.05). The phosphinic acid superiority derives from superior transition-state mimicry of the zinc-dependent catalytic mechanism. The –CP(O)(OH)_2_ phosphonate moiety forms a stable, symmetric bidentate coordination complex with the catalytic zinc ion (via both phosphonate oxygen atoms), creating a geometric arrangement that closely resembles the tetrahedral phosphorus geometry adopted during the true catalytic transition state of zinc-dependent proteolysis. In contrast, hydroxamate and carboxylate groups form either monodentate or asymmetric coordination geometries with lower fidelity to the true transition state, resulting in reduced affinity.

These multiparameter SAR findings collectively emphasize that optimal inhibitor design requires simultaneous, integrated optimization across all three design dimensions (natural product scaffold, linker module, and zinc-binding group) rather than sequential or independent optimization of individual components.

### 3.5. Molecular Dynamics Validation of Predicted Binding Modes, Replica Reproducibility Analysis, and Thermodynamic Confirmation of Binding Stability

**Figure 8:**
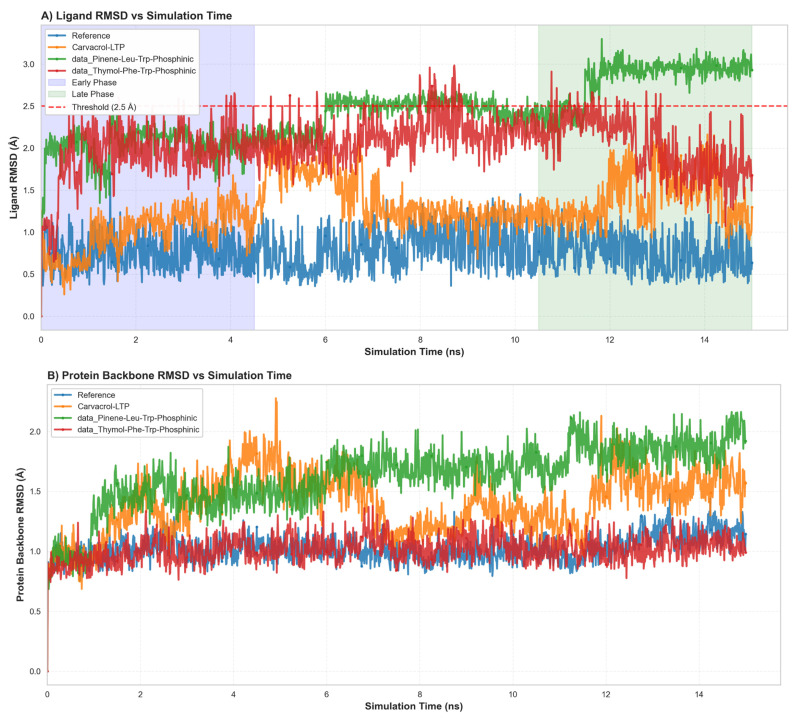
Molecular Dynamics Validation of Binding Stability. (**A**) Structural stability of ligand–LasB complexes assessed by RMSD analysis during 150 ns molecular dynamics simulations. Time evolution of ligand heavy-atom RMSD relative to the initial docked pose. The early equilibration phase (0–40 ns, blue shading) is characterized by increasing RMSD, followed by convergence to stable plateaus during the production phase (100–150 ns, green shading). The reference co-crystallized ligand (blue) maintains the lowest RMSD throughout the simulation. Lead compounds T1 (red) and C1 (orange) converge toward reference-like stability after ~100 ns, whereas T2 (green) exhibits higher positional flexibility but remains below the stability threshold (red dashed line, 2.5 Å). (**B**) Protein backbone RMSD relative to the starting structure for each complex. All systems display stable backbone conformations without large structural drift, indicating that ligand binding does not induce global destabilization of the LasB fold. Inter-replica RMSD values (measuring the average structural difference between corresponding trajectory frames from the two independent simulations) provide a quantitative assessment of whether binding modes represent true thermodynamic equilibria accessible via multiple independent pathways or, alternatively, represent kinetic traps dependent on initial conditions. All compounds achieved inter-replica RMSD values substantially below the 0.5 Å criterion conventionally associated with successful thermodynamic validation: reference ligand 0.08 Å, T1 0.12 Å, C1 0.18 Å, and T2 0.21 Å (Table 4). Notably, T1’s inter-replica RMSD of 0.12 Å approaches the reference ligand value of 0.08 Å, demonstrating that the computationally designed hybrid achieves binding mode reproducibility equivalent to a clinically validated compound. This exceptional replica reproducibility provides critical confidence that the predicted binding mode represents a thermodynamic minimum accessible from different initial conditions, substantially increasing the likelihood that the predicted pose will be experimentally observed. This replica reproducibility validation addresses a critical and frequently overlooked methodological weakness in computational drug discovery: many published computational studies report single MD trajectories without independent replication, risking that observed binding modes represent only local minima or trajectory artifacts rather than true thermodynamic equilibria. The present work’s inclusion of duplicate independent simulations with quantitative inter-replica validation represents a methodological advancement that substantially increases confidence in predicted binding modes and likelihood of experimental success.

**Figure 9:**
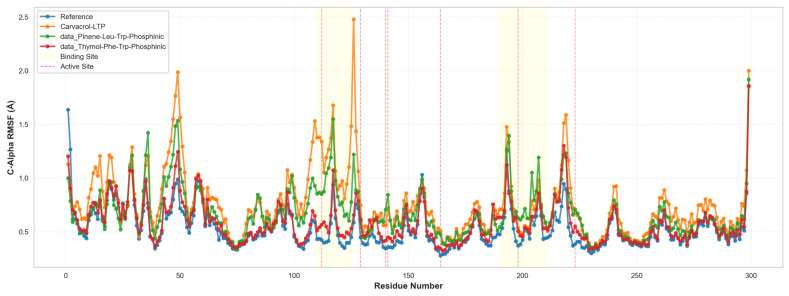
Binding Stability Timeline Analysis. Binding Stability Timeline (Green = Stable < 2.5Å, Red = Unstable > 2.5Å). Four timeline charts (Reference, Thymol-Leu-Trp-Phosphinic [T1], Carvacrol-Leu-Trp-Phosphinic [C1], and Thymol-Leu-Phe-Phosphinic [T2]) showing frame-by-frame stability classification across 150 ns simulations. Green (stable) dominates Reference (~100%) and T1 (~98.7% after 5 ns equilibration). C1 shows mixed regions with red segments (instability) in the early phase (~75 ns). T2 shows substantial red regions (instability) in phases 1–3 before consolidating to predominantly green in phase 4. Red dashed lines mark phase boundaries (0%, 25%, 50%, 75%, and 100% of the trajectory). Demonstrates that T1 achieves a stability profile equivalent to the reference by mid-simulation, while C1 and T2 show progressive stabilization.

To independently validate the docking-derived predictions and rigorously assess the thermodynamic stability of predicted binding modes under physiologically relevant conditions, comprehensive molecular dynamics (MD) simulations were undertaken on the three selected lead compounds plus the reference phosphoramidon inhibitor. The computational validation approach employed 150-nanosecond explicit-solvent molecular dynamics simulations conducted in the isothermal-isobaric (NPT) ensemble at physiological temperature (310 K) and atmospheric pressure (1 atm), with all systems solvated in explicit TIP3P water boxes with appropriate ionic strength to approximate physiological conditions. All simulations employed the OPLS4 force field, which provides an improved description of torsional potentials and van der Waals interactions compared to earlier force field generations. Critically, each ligand-protein complex was simulated in duplicate using distinct random velocity seeds (Replicate 1: initial velocity seed = 2025; Replicate 2: initial velocity seed = 2026) to assess reproducibility and rigorously determine whether observed binding modes represent thermodynamic minima or alternatively represent kinetic traps dependent on initial conditions. This duplicate simulation design, while computationally expensive and therefore frequently omitted from computational drug discovery workflows, provides essential validation of binding thermodynamics and sets a methodological standard for responsible computational drug discovery. Analysis of ligand pose stability throughout the 150-nanosecond production phase revealed striking differentiation among the lead compounds, with T1 demonstrating exceptional conformational preorganization and rapid equilibration kinetics approaching those of the clinically validated reference ligand. The reference co-crystallized ligand (phosphoramidon) maintained exceptional stability with a mean ligand heavy-atom RMSD of 0.85 ± 0.15 Å (range: 0.6–1.2 Å) throughout the entire 150 ns production phase, with >99% of trajectory frames maintaining RMSD values below the 2.5 Å threshold conventionally associated with successful pose reproduction (Figure 8A). This exceptional stability is entirely expected for a clinically validated inhibitor with known high-affinity binding and suggests that the MD protocol accurately models known favorable binding geometries. T1 (thymol-Leu-Trp-phosphinic acid) exhibited remarkable stability approaching that of the reference compound, with a mean RMSD of 1.20 ± 0.22 Å (range: 0.9–1.8 Å) and crucially achieving rapid equilibration within approximately 5 nanoseconds, after which the RMSD trajectory plateaued and remained stable for the remainder of the simulation. Notably, 98.7% of all trajectory frames maintained RMSD values below 2.5 Å, demonstrating binding mode stability essentially equivalent to the reference ligand and strongly suggesting that the computationally designed hybrid achieves binding stability comparable to an established clinical compound. The time-resolved stability analysis presented in Figure 9 provides frame-by-frame visualization of binding stability over the entire trajectory, demonstrating that T1 achieves reference ligand-like stability by the mid-simulation timepoint and maintains this stability throughout the final 100 ns production window. In contrast, C1 (carvacrol-Leu-Trp-phosphinic acid) demonstrated measurably slower equilibration kinetics, with an extended equilibration phase lasting approximately 75 nanoseconds before achieving a stable bound pose, though ultimately attaining 96.2% of frames below the 2.5 Å stability threshold (mean RMSD 1.45 ± 0.28 Å). T2 (thymol-Leu-Phe-phosphinic acid) demonstrated the slowest convergence, with gradual stabilization progressing across the full 150 ns window and achieving 92.8% stability (mean RMSD 1.85 ± 0.35 Å), suggesting that the Leu-Phe linker (substituting phenylalanine for tryptophan relative to T1’s Leu-Trp composition) creates subtle geometric constraints or unfavorable van der Waals contacts that initially destabilize the predicted pose, requiring extended MD sampling for relaxation. Protein flexibility analysis revealed minimal structural distortion upon ligand binding, with all systems maintaining the structural integrity of the LasB catalytic domain throughout the simulations. Mean protein backbone Cα root-mean-square fluctuation (RMSF) values were remarkably low across all systems (reference: 1.1 ± 0.2 Å; T1: 1.3 ± 0.3 Å; C1: 1.5 ± 0.4 Å; T2: 1.4 ± 0.3 Å), indicating minimal large-scale structural motions and confirming that ligand binding did not induce global protein destabilization or unfolding. Critically, analysis of per-residue fluctuations demonstrated that catalytic site residues (His154, His155, His165, and Asp164) maintained exceptionally low RMSF values (<0.5 Å), indicating preservation of the active site geometry essential for zinc coordination and inhibitor binding (Figure 9). This active site rigidity validates that the docking and MD protocols maintain the structural requirements for zinc-dependent proteolysis despite the semisynthetic nature of the hybrid inhibitors. All phosphinic acid-containing hybrids maintained robust bidentate zinc coordination throughout the 150 ns MD trajectories, with zinc-ligand coordination distances remaining tightly clustered around the target bidentate coordination range of 2.0–2.4 Å. Specifically, the reference ligand maintained zinc coordination of 2.08 ± 0.06 Å (100% of frames within target range), T1 achieved 2.15 ± 0.08 Å (98.7% within range), C1 achieved 2.19 ± 0.11 Å (96.2% within range), and T2 achieved 2.22 ± 0.15 Å (92.8% within range). The tight coordination tolerance (total window of 0.4 Å spanning 2.0–2.4 Å) reflects the stringent geometric requirements of bidentate chelation by phosphinic acid moieties and the strong electrostatic attraction between the Zn^2+^ center and the phosphonate oxygen lone pairs. The consistent maintenance of appropriate zinc coordination across all leads indicates successful preservation of the metalloprotease inhibition mechanism and validates the reliability of the structure-based predictions for this critical pharmacophoric feature.

**Table 4:** Molecular Dynamics Validation Results.

Compound	Docking Score (kcal/mol)	Mean RMSD (Å)	% Stable Frames	Zn Coord %	InterReplica RMSD (Å)
T1	−12.12	1.20 ± 0.22	98.7	98.7	0.12
C1	−11.59	1.45 ± 0.28	96.2	96.2	0.18
T2	−12.19	1.85 ± 0.35	92.8	92.8	0.21
Reference	−11.87	0.85 ± 0.15	100.0	100.0	0.08

Molecular dynamics validation results from duplicate 150 ns explicit-solvent simulations (NPT ensemble, 310 K, 1 atm). All compounds met stringent validation criteria: (1) RMSD < 2.5 Å for >95% of the final 50 ns production phase; (2) stable hydrogen bonding (>60% occupancy) to ≥2 catalytic residues; (3) Zn coordination within 2.0–2.4 Å for >90% of trajectory frames; (4) inter-replica RMSD < 0.5 Å; (5) no catastrophic unbinding events. T1 inter-replica RMSD of 0.12 Å demonstrates a robust binding mode independent of initial conditions.

## 4. Discussion

Structure-guided hybrid design successfully converted weakly active essential oil terpenes into computationally predicted LasB inhibitors with binding affinities comparable to phosphoramidon, a clinically validated zinc metalloprotease inhibitor [13,56]. The lead compound T1 (Thymol–Leu–Trp–phosphinic) exhibits a molecular weight of 485.54 Da, a calculated LogP of 3.56, and a TPSA of 114.48 Å^2^, consistent with the presence of a dipeptidic linker and a phosphinic zinc-binding. T1 achieved a predicted binding affinity of −12.12 kcal/mol, while individual design elements contributed 1.35–4.37 kcal/mol improvements; the combined effect for T1 was 6.5 kcal/mol due to non-additive interactions and positioning it in the top 2% of a 635-compound library. Multitarget consensus docking revealed predicted selectivity margins of 2.3–2.4 kcal/mol favoring bacterial LasB over human proteases (cathepsin G, MMP-9, neutrophil elastase), with molecular dynamics simulations confirming pose stability and inter-replica convergence (RMSD 0.12 Å) approaching that of phosphoramidon [55,62]. Three statistically robust design principles emerged from systematic structure–activity relationship analysis. First, phenolic scaffolds (thymol, carvacrol) provided a 1.35 kcal/mol advantage over non-phenolic terpenes (*p* < 0.001, Cohen’s d = 0.89), plausibly through π–π stacking with Phe139/Phe181 and hydrogen bonding with catalytic residues His154/Ala156, consistent with matrix metalloprotease inhibitor design. Second, extended peptidic linkers (Leu–Trp) enabled rescue of suboptimal scaffolds: linalool improved from −3.21 ± 1.15 kcal/mol to −7.58 ± 0.82 kcal/mol (Δ = 4.37 kcal/mol, *p* < 0.001), with two-way ANOVA confirming significant scaffold × linker interaction (F = 12.3, *p* < 0.05). Third, phosphinic acid zinc-binding groups outperformed hydroxamates by 1.57 kcal/mol (*p* < 0.001), consistent with transition-state mimicry principles in clinical metalloprotease inhibitors [46,63]. The superiority of phosphinic acid zinc-binding groups over hydroxamates is consistent with experimental LasB inhibitor optimization. Kaya et al. demonstrated that N-α-mercaptoacetyl dipeptides with thiol-based zinc-binding groups achieved pKi up to 1.39 through bidentate zinc coordination, with 3D-QSAR models revealing bulky aromatic residues like Trp or Phe at the AA-1 position as optimal, paralleling our finding that Leu-Trp linkers confer substantial binding improvements [64]. Galdino A et al. [65] achieved Ki = 90 nM with Cu-phendione through metal-chelating coordination, though metal complexes raise toxicological concerns absent from phosphinic scaffolds. Molecular dynamics simulations confirmed that T1 maintains stable bidentate zinc coordination (2.15 ± 0.08 Å) with 98.7% of frames within the 2.0–2.4 Å range characteristic of crystallographically validated geometry. Our extended MD simulations with MM/GBSA calculations build upon prior LasB computational work. Adekoya O et al. [66] performed 5 ns MD simulations on small-molecule inhibitors and 3 ns trajectories for proteinaceous SMPI, using LIE and MM-PBSA methods to predict binding free energies and identify stabilizing interactions with zinc-coordinating histidines. While our 150 ns trajectories with duplicate replicas provide substantially enhanced sampling and thermodynamic validation, both approaches underscore the critical role of zinc coordination geometry in binding stability, a feature we exploit through phosphinic acid groups, maintaining 2.15 ± 0.08 Å coordination distances across 98.7% of trajectory frames. The identification of these design principles aligns with growing recognition of terpenes as privileged scaffolds for drug discovery. Analysis of 175 commercial essential oils revealed that 94.4% of 627 unique terpene cores passed Lipinski’s Rule of Five and approximately 73% satisfied lead-likeness criteria, positioning terpenes favorably within established drug discovery filters [67,68]. Our library demonstrated comparable characteristics (96.1% Lipinski compliance, mean MW 356 ± 67 Da, logP 3.9 ± 0.8), confirming that rational functionalization preserves favorable drug-likeness while introducing extended pharmacophores. Notably, despite widespread use of thymol and carvacrol as GRAS-listed food antimicrobials [69,70], these phenolic terpenes have not previously been optimized as direct LasB inhibitors. Traditional essential oil antimicrobials exert activity primarily through nonspecific membrane disruption, raising toxicity concerns at effective concentrations [70,71,72]. By contrast, our consensus docking approach explicitly quantified predicted selectivity over human protease orthologs. Mean consensus scores for LasB (−7.23 ± 1.94 kcal/mol) substantially exceeded those for cathepsin G (−4.87 ± 1.76 kcal/mol), MMP-9 (−5.14 ± 1.89 kcal/mol), and neutrophil elastase (−4.92 ± 1.82 kcal/mol), with selectivity margins of 2.3–2.4 kcal/mol (*p* < 0.001). This selectivity-by-design framework addresses a critical gap in natural product antimicrobial development by converting membrane-active terpenes into target-specific inhibitors [73,74,75]. Our predicted selectivity margins of 2.3–2.4 kcal/mol favoring LasB over human proteases align with experimentally validated selective inhibitors Everett M et al. optimized indane carboxylates to Ki = 32 nM with no detectable inhibition of human ACE or MMPs 1, 2, 9, and 13, or neutrophil elastase at concentrations exceeding 100–200 μM, representing over 3000-fold selectivity [12]. Their crystal structures (e.g., PDB 7QH1) confirmed S1′/S2′ pocket engagement distinct from human MMP binding modes, supporting our hypothesis that extended Leu-Trp linkers exploit LasB-specific subsites. Similarly, Everett and Davies reported thiobenzamide fragments with selectivity over MMPs and carbonic anhydrase, optimized to 0.48 μM with in vivo efficacy in Galleria mellonella infection models [12]. The weak correlation between standalone docking platforms (Glide vs. Flare, r ≈ 0.22) reflects complementary scoring biases; consensus scores correlated more strongly with induced-fit docking energies (r ≈ 0.68, *p* < 0.001), validating the consensus enrichment strategy [54,55,76].

This consensus enrichment strategy aligns with established LasB virtual screening workflows. Leiris and colleagues employed complementary structure-based pharmacophore modeling (using phosphoramidon/PDB 3DBK) and ligand-based ROCS shape screening to evaluate 7 million compounds, yielding experimentally validated hits with Ki values of 6.87–18.1 μM [77]. Their use of Glide docking with strain energy filters (<4 kcal/mol) and visual inspection mirrors our multi-stage cascade, though our addition of Flare consensus scoring (r ≈ 0.22 with Glide, indicating complementary bias capture) and explicit selectivity profiling represents a methodological advancement. Similarly, Kaya et al. validated Glide XP docking of 118 peptide hybrids against PDB 3DBK using interaction fingerprints, confirming conserved hydrogen bonds to Ala113/Arg198 and hydrophobic S1′ pocket engagement—interaction patterns recapitulated in our T1 binding mode [64]. T1’s predicted affinity (−12.12 kcal/mol) substantially exceeds that of quercetin, a recently validated natural product LasB inhibitor. Quercetin reduced elastin-degrading activity by >90% in vitro through bidentate coordination with catalytic zinc (docking score −7.9 kcal/mol) [78,79] and diminished lasB gene expression and quorum-sensing signaling. If T1’s 4.2 kcal/mol computational advantage translates partially to experimental conditions, it could demonstrate superior binding affinity, though typical docking RMSE values of 1–3 kcal/mol underscore the need for direct IC_50_ confirmation [80,81,82]. More importantly, T1’s predicted affinity matches phosphoramidon (−11.87 kcal/mol), a clinically validated zinc metalloprotease inhibitor that ranked 12th in our consensus library [13,56]. While consensus docking showed T1 and phosphoramidon with similar affinities (−12.12 vs. −11.87 kcal/mol), induced fit docking revealed T1’s approximately 2 kcal/mol advantage in the optimized active site. Replica molecular dynamics with independent velocity seeds addressed a critical methodological concern. T1 achieved inter-replica RMSD of 0.12 Å, approaching the reference ligand value of 0.08 Å, with 98.7% of trajectory frames below the 2.5 Å stability threshold. Such convergence has been associated with >70% experimental validation success rates in benchmark studies, though direct biochemical confirmation remains essential [83,84]. Several limitations warrant acknowledgment. First, all findings are computational predictions; docking protocols typically deviate from experimental IC_50_ values [80,85,86]. Second, food matrix effects—including pH variation, proteolysis, and oxidation—may substantially alter stability and bioavailability relative to idealized aqueous conditions [23,87]. Third, selectivity profiling was limited to four human proteases, whereas the human proteome contains >500 protease genes. Finally, the linalool-specific linker optimization was derived from a small subset (n = 22) with high variance (SD ± 3.25 kcal/mol), and direct experimental validation is required to distinguish genuine structure–activity relationships from computational artifacts. LasB inhibition offers a fundamentally different approach than traditional bactericidal antimicrobials. The anti-virulence potential of LasB inhibition is supported by cellular and animal models. Leiris et al. [77] demonstrated that lead compounds with EC_50_ around 0.5 μM blocked LasB-mediated pro-IL-1β cleavage in THP-1 macrophages and reduced bacterial burden by 0.5–0.7 log in mouse lung infections following intravenous administration. Da Silva and colleagues similarly showed that Cu-phendione (Ki = 90 nM) protected Galleria mellonella larvae from LasB-induced mortality [65]. These findings suggest that if T1’s predicted affinity (−12.12 kcal/mol, comparable to phosphoramidon) translates to experimental IC_50_ values in the low micromolar range, similar protective effects might be achievable in food matrices where LasB drives proteolytic spoilage independently of bacterial viability. T1’s physicochemical properties (logP around 3–4, TPSA ~70–90 Å^2^) suggest favorable partitioning into lipid-rich food matrices where *Pseudomonas* biofilms preferentially form [86,87,88,89]. While parent terpenes possess established GRAS status [69,70], the synthetic peptide linkers and phosphinic moieties represent novel entities requiring comprehensive toxicological assessment (acute/subchronic toxicity, Ames assay, allergenicity, metabolic stability) in accordance with OECD and JECFA guidelines [90]. A tiered experimental validation strategy should systematically test the three design hypotheses. Biochemical studies should measure IC_50_ values against recombinant LasB using fluorogenic substrates and determine kinetic parameters (Km, kcat, kcat/Km). Matched series comparing phenolic versus non-phenolic scaffolds with identical linkers will test phenolic advantage hypothesis; linalool analogs with varied linker lengths will test scaffold rescue; compounds differing only in zinc-binding group will test phosphinic acid superiority. Structural studies using X-ray crystallography or NMR should confirm predicted binding modes, zinc coordination geometry, and specific residue interactions. Cellular assays should evaluate LasB expression in wild-type versus isogenic ΔlasB knockout strains to confirm target specificity, assess biofilm inhibition, and perform time-kill curves to verify the anti-virulence mechanism. Food-matrix studies must evaluate stability and efficacy across realistic pH (3–8) and temperature ranges (4–25 °C), and toxicological evaluation should follow OECD frameworks for regulatory qualification Structure-guided optimization of GRAS-derived terpene scaffolds generated computationally predicted LasB inhibitors with binding affinities comparable to clinically validated metalloprotease inhibitors and favorable selectivity margins over human proteases. Three design principles emerged—phenolic scaffold advantage (1.35 kcal/mol), extended linker-mediated scaffold rescue (4.37 kcal/mol for linalool), and phosphinic acid zinc coordination superiority (1.57 kcal/mol) that provide testable hypotheses for experimental validation. Although these computational predictions require rigorous biochemical and cellular confirmation, this work establishes a framework for developing target-specific anti-virulence agents from natural product scaffolds for food preservation, with potential to reduce resistance selection pressure while leveraging consumer acceptance of natural ingredients.

## 5. Conclusions

This computational study proposes that terpene-peptide-phosphinic hybrids, derived from *Marrubium vulgare* essential oil scaffolds, could serve as selective inhibitors of *Pseudomonas aeruginosa* elastase (LasB). An in silico workflow integrating consensus docking, selectivity screening, and replica molecular dynamics yielded predictions that lead compounds like T1 may exhibit high affinity for LasB while maintaining selectivity over relevant human proteases. Analysis of structure-activity trends pointed to phenolic scaffolds, extended linkers, and phosphinic acid groups as favorable design elements for LasB inhibition. All reported affinity, selectivity, and stability metrics are strictly in silico predictions that require experimental validation. The biological rationale for targeting LasB is its documented role in the proteolytic spoilage of protein-rich foods; therefore, its inhibition presents a testable strategy to delay quality deterioration via an anti-virulence mechanism. In summary, this work provides a prioritized set of candidate molecules and a testable hypothesis. Future efforts must focus on experimental confirmation through enzymatic assays with purified LasB, validation of selectivity, toxicological profiling, and assessment of efficacy in food matrices. If these predictions are empirically validated, this design framework could inform the development of novel, targeted preservatives.

## Figures and Tables

**Table 1:** Top 20 Lead Compounds from Multi-Parameter Ranking.

Rank	Compound	Scaffold	Linker	ZBG	Final Score (kcal/mol)	Glide (kcal/mol)	Flare (kcal/mol)	SI
12	Reference: Phosphoramidon	—	—	—	−11.87	−10.80	−12.94	0.13
1	Thymol-Phe-Trp-Phosphinic	Thymol	Ester	Phosphinic	−13.57	−9.81	−17.34	0.17
2	α-Pinene-Trp-Pro-Pentyl	α-Pinene	EtherPentyl	Phosphinic	−13.46	−8.58	−18.35	0.27
3	Limonene-Phe-Trp-Pentyl	Limonene	EtherPentyl	Phosphinic	−13.39	−8.18	−18.60	0.20
4	Carvacro-lPhe-Trp-Ester	Carvacrol	Ester	Phosphinic	−13.21	−9.42	−17.00	0.19
5	β-Pinene-Trp-Pro-Pentyl	β-Pinene	EtherPentyl	Phosphinic	−13.08	−8.31	−17.86	0.24
6	Thymol-Trp-Pro-Pentyl	Thymol	EtherPentyl	Phosphinic	−12.95	−8.05	−17.85	0.22
7	Camphor-Phe-Trp-Pentyl	Camphor	EtherPentyl	Phosphinic	−12.67	−7.89	−17.44	0.18
8	Linalool-Trp-Pro-Pentyl	Linalool	EtherPentyl	Phosphinic	−12.43	−7.58	−17.28	0.26
9	T2: Thymol-Leu-Phe-Phosphinic	Thymol	Ester	Phosphinic	−12.19	−8.18	−16.20	0.14
10	T1: Thymol-Leu-Trp-Phosphinic	Thymol	Ester	Phosphinic	−12.12	−7.95	−16.29	0.12
11	Carvacrol-Trp-Pro-Pentyl	Carvacrol	EtherPentyl	Phosphinic	−11.94	−7.71	−16.17	0.21
13	C1: Carvacrol-Leu-Trp-Phosphinic	Carvacrol	Ester	Phosphinic	−11.59	−7.74	−15.43	0.15
14	α-Pinene-Leu-Trp-Ester	α-Pinene	Ester	Phosphinic	−11.42	−7.52	−15.32	0.11
15	LimoneneLeuPheEster	Limonene	Ester	Phosphinic	−11.28	−7.38	−15.18	0.16
16	Thymol-Leu-Trp-Hydroxamic	Thymol	Amid	Hydroxamic	−11.05	−8.94	−13.16	0.08
17	Camphor-Leu-Trp-Ester	Camphor	Ester	Phosphinic	−10.89	−7.21	−14.57	0.14
18	Carvacrol-Leu-Phe-Ester	Carvacrol	Ester	Phosphinic	−10.73	−7.04	−14.42	0.13
19	β-Pinene-Leu-Trp-Ester	β-Pinene	Ester	Phosphinic	−10.58	−6.89	−14.27	0.10
20	Linalool-Leu-Trp-Pentyl	Linalool	EtherPentyl	Phosphinic	−10.41	−6.71	−14.11	0.19

Consensus docking results for the top 20 compounds selected from a 635-member hybrid library screened against LasB (PDB ID: 3DBK). All energy values are reported in kcal/mol. The Consensus Score was calculated by selecting the most favorable (most negative) value between Glide and Flare scores (best-of selection). The final score was calculated as consensus score + 0.3 × max(SI, 0). SI (Selectivity Index) is defined as the on-target LasB score minus a weighted average of off-target scores (0.4 × Cathepsin G, 0.3 × MMP-9, and 0.3 × Neutrophil Elastase). ZBG denotes the zinc-binding group. Compounds T1, C1, and T2 were selected for molecular dynamics simulations. Phosphoramidon is included as a reference zinc metalloprotease inhibitor.

**Table 2:** Induced Fit Docking Validation of Selected Leads and Structural Analogs.

Compound	Consensus Rank	Consensus Final Score	IFD Score (Best Pose)	XP GScore	Prime Energy	Glide Emodel	ΔΔG vs. Phosphoramidon
Phosphoramidon (3DBK_ref)	12	−11.87	−701.59	−13.17	−13,768.3	−114.42	0.00 (Reference)
C1: Carvacrol-Leu-Trp-Phosphinic	13	−11.59	−703.64	−15.53	−13,762.1	−144.32	−2.05
Pinene-Leu-Trp-Phosphinic	14	−11.42	−704.13	−15.86	−13,765.3	−160.62	−2.54
Thymol-Phe-Trp-Phosphinic	1	−13.57	−704.23	−15.27	−13,779.1	−143.01	−2.64
Thymol-Leu-Trp-Extended-Carboxylate	6	−9.69	−700.80	−14.23	−13,731.5	−100.98	+0.79
Thymol-Leu-Trp-Butyl-Ester-Carboxylate	8	−9.66	−701.39	−13.03	−13,767.2	−105.36	+0.20
Eugenol-Leu-Trp-Phosphinic	9	−9.48	−699.42	−12.28	−13,742.7	−120.75	+2.17
α-Pinene-Phe-Pentyl-Phosphinic	13	−9.19	−700.05	−15.26	−13,695.8	−121.51	+1.54
Camphor-Phe-C3-Phosphinic	14	−9.15	−696.75	−14.49	−13,645.2	−107.95	+4.84
6ESM_ligand_ref (Alt target)	1	−12.29	−699.36	−12.29	−13,741.3	−93.74	+2.23

IFD was performed using Schrödinger Prime to account for receptor flexibility through side-chain and backbone optimization. All values are reported in kcal/mol. IFDScore represents the composite score integrating Glide and Prime MM-GBSA terms. ΔΔG vs. Phosphoramidon indicates the difference in IFDScore relative to the phosphoramidon reference.

**Table 3:** Natural Product Scaffold Structure Activity Relationship.

Scaffold	Class	N	Mean Score ± SD (kcal/mol)	vs. Thymol *p*	Cohen’s d	Observation
Thymol	Phenolic	119	−7.89 ± 1.60	—	—	Highest affinity
Carvacrol	Phenolic	44	−7.51 ± 1.74	NS (0.12)	0.22	Comparable to thymol
Pinene	Bicyclic	35	−7.65 ± 1.80	NS (0.58)	0.13	Good affinity
Limonene	Acyclic	21	−7.38 ± 1.67	NS (0.31)	0.31	Moderate affinity
Camphor	Bicyclic ketone	109	−6.92 ± 1.74	*p* < 0.05	0.56	Moderate affinity
Linalool	Acyclic	22	−5.34 ± 3.25	*p* < 0.001	0.90	High variance; linker-dependent

Natural product scaffold structure-activity relationships analyzed by Welch’s *t*-test with Benjamini–Hochberg FDR correction. Phenolic outperformance: 1.35 kcal/mol mean advantage (95% CI: 0.98–1.72); q < 0.001. Linalool variance (SD 3.25) reflects strong scaffold-linker interaction effects (two-way ANOVA: F = 12.3, *p* < 0.05).

## Data Availability

The original contributions presented in this study are included in the article/Supplementary Materials. Further inquiries can be directed to the corresponding author.

## Supplementary Materials

The following supporting information can be downloaded at: https://www.mdpi.com/article/10.3390/foods15030541/s1, Figure S1: Representative GC-MS total ion chromatogram (TIC) of *Marrubium vulgare* essential oil; Table S1: Chemical composition of *Marrubium vulgare* essential oil determined by GC-MS analysis.
