# Supplementary material for: Rational Design and Virtual Screening of Antimicrobial Terpene-Based Leads from Marrubium vulgare Essential Oil: Structure-Based Optimization for Food Preservation and Safety Applications
Source: Foods. 2026 Feb 4;15(3):541. doi: 10.3390/foods15030541 (PMC12897207; doi:10.3390/foods15030541)

Supplementary Data File S1

Rational Design and Virtual Screening of Antimicrobial TerpeneBased Leads from Marrubium vulgare Essential Oil: StructureBased Optimization for Food Preservation and Safety Applications

S1.1. Botanical Identification

Scientific name: *Marrubium vulgare* L.

Common names: White horehound, Common horehound

Family: Lamiaceae

Subfamily: Lamioideae

Authority: Linnaeus, Species Plantarum 2: 583. 1753

Taxonomic Authentication:
Plant identification and taxonomic authentication were performed by certified botanists, including Prof. Mohamed Chaieb, PhD & Doctor of State, at the Laboratory of Biodiversity and Ecosystems in Arid Environments (LEBIOMAT), Faculty of Sciences, University of Sfax, Sfax, 3000, Tunisia.

Voucher Specimen:
A herbarium voucher specimen was prepared and deposited to allow future verification and reference.

S1.2. Collection Site Details

Geographic location:

- Country: Tunisia
- Region: Nabeul Governorate
- Locality: Cité Hached, Bouargoub
- GPS Coordinates: 36°30'27.5"N, 10°33'39.8"E
- Elevation: Approximately 15–20 m above sea level

Habitat characteristics:

- Climate type: Mediterranean climate with hot, dry summers and mild, wet winters
- Vegetation type: Semi-arid Mediterranean scrubland
- Soil type: Calcareous soil typical of coastal Tunisian regions

Collection date: Spring 2024 (March–May 2024)

Phenological stage: Vegetative growth to early flowering stage

Collection time: Early morning hours (07:00–09:00 AM local time) to minimize volatile compound loss due to heat and sunlight exposure

Weather conditions: Typical spring conditions with moderate temperatures (18–27°C) and low to moderate humidity

S1.3. Plant Material Characteristics

Plant parts collected: Aerial parts (leaves and flowering tops)

Health status: Healthy plants free from visible disease, pest damage, or physical injury were selected for collection

Moisture content before drying: Approximately 75–80% (typical for fresh plant material)

Final moisture content after drying: <10% w/w (determined by loss-on-drying method at 105°C until constant weight)

S1.4. Post-Harvest Handling

Transportation: Fresh plant material was transported to the laboratory within 2 hours of collection in breathable cotton bags to prevent moisture accumulation and microbial contamination.

Cleaning procedure: Upon arrival at the laboratory, plant material was carefully inspected and cleaned manually to remove:

- Soil particles and dust
- Damaged or discolored leaves
- Foreign plant material
- Any contaminants

Drying conditions:

- Method: Air-drying in the shade
- Location: Well-ventilated laboratory room away from direct sunlight
- Temperature: 22 ± 2°C
- Relative humidity: 40–50%
- Duration: 12–15 days
- Final state: Leaves became brittle and easily crumbled when rubbed

Storage before extraction: Dried plant material was stored in paper bags at room temperature in a dark, dry place until essential oil extraction.

S1.5. Essential Oil Extraction Protocol

Extraction method: Hydrodistillation using a Clevenger-type apparatus

Equipment specifications:

- Apparatus: Clevenger-type apparatus (all-glass design)
- Heating source: Electric heating mantle with temperature control
- Flask volume: 1000 mL round-bottom flask
- Condenser: Water-cooled condenser

Extraction parameters:

- Plant material mass: 100 g of dried aerial parts per extraction
- Water volume: 500 mL distilled water
- Extraction time: 3 hours from the beginning of boiling
- Temperature: Maintained at gentle boiling (~100°C at atmospheric pressure)
- Atmospheric pressure: ~1013 mbar (sea level)
- Number of replicates: 3 independent extractions

Post-distillation processing:

1. **Collection:** Essential oil was collected from the graduated tube of the Clevenger apparatus
2. **Drying:** Residual water was removed using anhydrous sodium sulfate (Na_2SO_4, ACS grade)
3. **Liquid-liquid extraction:** Oil was further purified by liquid-liquid extraction with n-hexane (HPLC grade, Sigma-Aldrich, St. Louis, MO, USA)
4. **Concentration:** Hexane was removed using a rotary evaporator (Heidolph Laborota 4000, Schwabach, Germany) under reduced pressure (−0.8 bar) at 35°C
5. **Final collection:** Pure essential oil was collected in amber glass vials (10 mL capacity)
6. **Storage:** Vials were sealed with PTFE-lined caps and stored at 4°C in darkness until GC-MS analysis

Extraction yield:

The extraction yield was calculated using the following formula:

Yield (%) = (Mass of essential oil (g) / Mass of dry plant material (g)) × 100

All extractions were performed in triplicate to ensure reproducibility.

Physical characteristics of the essential oil:

- Appearance: Clear to pale yellow liquid
- Odor: Characteristic aromatic, herbaceous odor typical of Lamiaceae family

S1.6. GC-MS Analysis Conditions

Instrument: Agilent 6890N gas chromatograph coupled with 5973 mass selective detector (Agilent Technologies, Palo Alto, CA, USA)

Column: HP-5MS fused silica capillary column (30 m × 0.25 mm, 0.25 µm film thickness; Agilent 19091S-433), coated with 5% phenyl methyl siloxane

Injection parameters:

- Sample preparation: 1:10 (v/v) dilution in analytical-grade n-hexane
- Injection volume: 1.0 µL
- Injection mode: Split mode (split ratio 30:1)
- Injector temperature: 250°C

Carrier gas:

- Type: Helium (purity ≥99.999%)
- Flow rate: 0.9 mL/min (constant flow mode)
- Average linear velocity: 34.6 cm/s
- Column head pressure: 6.5 psi

Oven temperature program:

- Initial temperature: 50°C (hold 1 min)
- Ramp rate: 6°C/min
- Final temperature: 250°C (hold 3 min)
- Total run time: 37.3 min

Mass spectrometer parameters:

- Ionization mode: Electron impact (EI)
- Ionization energy: 70 eV
- Transfer line temperature: 280°C
- Ion source temperature: 230°C
- Quadrupole temperature: 150°C
- Acquisition mode: Full scan
- Mass range: m/z 50–550
- Solvent delay: 3.00 min
- Gain factor: 1.0
- Electron multiplier voltage: ~1624 V

Compound identification:

2. Mass spectral comparison with NIST and Wiley 9th Edition spectral libraries (match quality ≥85%)
3. Calculation of Kovats' retention indices (RIs) using a homologous series of n-alkanes

Peak #	Retention Time (min)	Peak Type	Peak Width (min)	Peak Area	Start Time (min)	End Time (min)	Compound Name	Relative Abundance (%)[a]	Identification Method
1	7.175	VB	0.030	1,124,236	7.135	7.227	β-Pinene	1.67	RI + MS
2	8.383	BV	0.030	20,972,895	8.326	8.428	dl-Limonene	31.22	RI + MS
3	10.069	BB	0.031	2,708,949	10.019	10.147	Linalool	4.03	RI + MS
4	12.283	BB	0.038	1,415,108	12.178	12.364	α-Terpineol	2.11	RI + MS
5	13.764	BB	0.032	32,681,001	13.698	13.894	Linalyl acetate	48.65	RI + MS
6	16.186	BB	0.035	1,537,941	16.139	16.282	Neryl acetate	2.29	RI + MS
7	16.340	BB	0.033	1,951,417	16.292	16.411	4-tert-Butylcyclohexyl acetate	2.90	RI + MS
8	16.599	BB	0.033	2,187,697	16.554	16.711	Geranyl acetate	3.26	RI + MS
9	17.793	BB	0.034	1,038,592	17.717	17.874	trans-α-Bergamotene	1.55	RI + MS
10	19.284	BB	0.035	1,565,727	19.237	19.385	β-Bisabolene	2.33	RI + MS
				67,183,563			Total identified	100.00	

(C8–C24) analyzed under identical chromatographic conditions

S1.7. Chemical Composition of *M. vulgare* Essential Oil

Table S1. Chemical composition of *Marrubium vulgare* essential oil determined by GC-MS analysis

[a] Relative abundance calculated as: (Individual peak area / Total peak area) × 100

Peak type abbreviations: VB = Valley to baseline; BV = Baseline to valley; BB = Baseline to baseline

RI = Retention Index (Kovats Index); MS = Mass Spectrum comparison with NIST/Wiley libraries

S1.8. Supplementary Figure

Figure S1. Representative GC-MS total ion chromatogram (TIC) of *Marrubium vulgare* essential oil showing the separation and relative abundance of major terpene constituents. Peak numbers correspond to compounds listed in Table S1. The chromatogram displays excellent baseline resolution with the two major peaks at 13.764 min (linalyl acetate, 48.65%) and 8.383 min (dl-limonene, 31.22%) clearly dominating the profile.

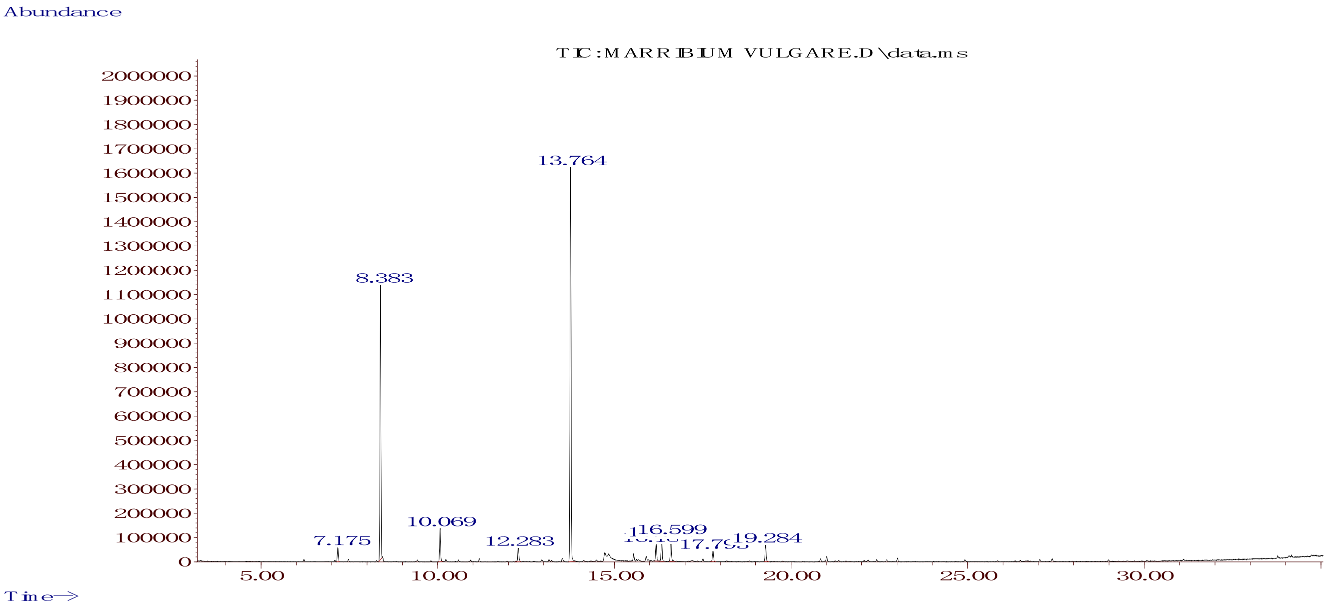

Supplement: Supplementary file 1 [file foods-15-00541-s001.zip › foods-4105032-supplementary.pdf]
